# RSAD2/VIPERIN and CMPK2 coordinate an immunometabolic response to Epstein-Barr Virus

**DOI:** 10.1371/journal.ppat.1013973

**Published:** 2026-08-03

**Authors:** Urvi S. Zankharia, Adam M. Glass, Wujuan Zhang, Qing Zhu, Janvhi Suresh Machhar, Ying Ye, Bhanu Chandra Karisetty, Jayamanna Wickramasinghe, Andrew Kossenkov, Jozef Madzo, Sun Sook Chung, Samantha S. Soldan, Richard Jason Lamontagne, James M. Wood, Lawrence D. Harris, Tyler L. Grove, Steven Jacobson, Aaron R. Goldman, Chengyu Liang, Paul M. Lieberman

**Affiliations:** 1 The Wistar Institute, Philadelphia, Pennsylvania, United States of America; 2 Victoria University of Wellington, Wellington, New Zealand; 3 Albert Einstein School of Medicine, New York, New York, United States of America; 4 NINDS, NIH, Bethesda, Maryland, United States of America; University of Southern California, UNITED STATES OF AMERICA

## Abstract

Epstein-Barr Virus (EBV) infection and reactivation in B-lymphocytes are tightly regulated by host antiviral response genes. In the present study, we identify Interferon Stimulated Genes (ISGs) RSAD2 (radical S-adenosyl methionine domain-containing 2) and CMPK2 (Cytidine/Uridine Monophosphate Kinase 2) as key modulators of EBV expression and cellular response during EBV infection and reactivation. EBV primary infection and reactivation lead to a coordinated upregulation of RSAD2 and CMPK2. Depletion of RSAD2 reduced cell viability and limited EBV reactivation, while depletion of CMPK2 led to reactivation of EBV lytic gene expression during latency. Despite distinct subcellular localizations, RSAD2 at the endoplasmic reticulum (ER) and CMPK2 in the mitochondria, transcriptomic analysis revealed that both genes functionally converge and exhibit overlapping roles in driving shared immunometabolic pathways, specifically Interferon (IFN) signaling, MAPK signaling, oxidative phosphorylation, mitochondrial function, eukaryotic translation, and ATF-4-associated unfolded protein response (UPR). We show that RSAD2 and CMPK2 knockdown affects IRAK1-TRAF6-TAK1 expression levels, and RSAD2 overexpression downregulates NF-κB signaling by EBV membrane associated oncoprotein LMP1. Depletion of RSAD2 and CMPK2 had significant effects on global metabolites consistent with a remodeling of nucleotide metabolism, glycolysis, fatty acid biosynthesis and degradation of superoxides. EBV reactivation induced formation of antiviral ribonucleotide ddhCTP which was strictly dependent on RSAD2. These observations demonstrate that RSAD2 and CMPK2 function in a coordinated ER-Mitochondria-Interferon signaling axis that shapes EBV reactivation and host immune control, including a novel layer of immunometabolic regulation modulating viral latency and reactivation.

## Author summary

Understanding how Epstein-Barr virus (EBV) regulates host factors to control infection, latency and reactivation is critical for developing targeted therapies against EBV-associated diseases. This study identifies Interferon-Stimulated Genes (ISGs) RSAD2 (Viperin) and CMPK2 as key regulators of EBV reactivation and host interferon responses in B-cells. Despite distinct organelle localizations, both genes converge on shared immunometabolic pathways, revealing a coordinated ER-Mitochondria axis that shapes viral expression and immune signaling. These findings provide new insights into the roles of host antiviral effectors and uncover potential targets for modulating EBV activity in inflammatory and oncogenic contexts.

## Introduction

Epstein–Barr virus (EBV), a member of the γ-herpesvirus family, persistently infects more than 90% of the global population and is implicated in a range of malignancies including B-cell lymphomas, nasopharyngeal carcinoma and gastric carcinoma, collectively accounting for over 200,000 cases of cancer annually [1,2]. Beyond oncogenesis, EBV infection has been strongly associated with autoimmune disorders such as Multiple Sclerosis (MS), where it is considered an essential cofactor [3]. A critical yet incompletely understood aspect of EBV biology is the role of interferon (IFN) signaling and interferon-stimulated genes (ISGs) in regulating viral latency, lytic reactivation, and host immune response. IFN-signaling induces a broad repertoire of ISGs that establish an antiviral state by restricting viral replication at multiple stages of the viral life cycle [4]. How IFN response controls EBV infection and may contribute to latency establishment is not completely understood.

RSAD2 (encoding Viperin, Virus Inhibitory Protein Endoplasmic Reticulum Associated Interferon-Inducible) and CMPK2 (Cytidine/Uridine Monophosphate Kinase 2) are two ISGs located immediately adjacent to each other on chromosome 2p25 and cotranscribed during IFN- signaling [5]. Both are expressed as a fusion protein in some lower organisms [6]. RSAD2/Viperin is highly evolutionarily conserved radical S-adenosylmethionine (SAM) enzyme with broad-spectrum antiviral activity. RSAD2/Viperin catalyzes the conversion of cytidine triphosphate (CTP) into 3′-deoxy-3′,4′-didehydro-CTP (ddhCTP), a nucleotide analog that acts as a chain terminator for viral RNA-dependent RNA polymerases, thereby inhibiting replication of viruses such as Zika virus, West Nile Virus and SARS-CoV2 [6–8]. Beyond its antiviral role, RSAD2 is increasingly recognized as a biomarker in autoimmune diseases, including Systemic Lupus Erythematosus (SLE) [9], Rheumatoid Arthritis [10] and in various cancers, where its expression correlates with poor prognosis [10,11]. RSAD2 is highly expressed in mouse dendritic cells (mDCs) and is necessary for IRF-7 mediated maturation of mDCs [12]. Depletion of RSAD2 led to loss of mDCs’ ability to induce proinflammatory cytokine production and T-cell proliferation in pulmonary metastasis [12]. CMPK2, a mitochondrial nucleotide kinase, catalyzes the phosphorylation of cytidine and uridine monophosphates to their diphosphate forms, providing essential precursors for mitochondrial DNA synthesis [1,5]. CMPK2 is crucial for maintenance of macrophage homeostasis and polarization [13]. Overexpression of CMPK2 promotes microglial activation and neuroinflammation through cGAS-STING pathway, implicating it in neurodegenerative disease mechanisms [14].

Despite these insights, the roles of RSAD2 and CMPK2 in EBV infection and host gene regulation remain poorly understood. We show here that RSAD2 and CMPK2 are coordinately regulated during EBV infection and reactivation in B-lymphocytes. To elucidate their function in EBV biology, we employed lentiviral-mediated knockdown of RSAD2 and CMPK2 in EBV+ Burkitt Lymphoma (BL) cell lines to assess their impact on viral gene expression and host immune response. We found that RSAD2 and CMPK2 orchestrate distinct yet overlapping roles in regulating EBV reactivation and host innate immune responses. Their knockdown disrupts IFN response, upregulates pathways and metabolites involved in mitochondrial metabolism and alters unfolded protein response dynamics, highlighting a coordinated ER-mitochondria-immune axis that shapes EBV pathogenesis and host defense.

## Results

### RSAD2 and CMPK2 are upregulated during EBV primary infection, lytic reactivation and in MS-patient derived SLCLs with unstable latency

**Fig 1:**
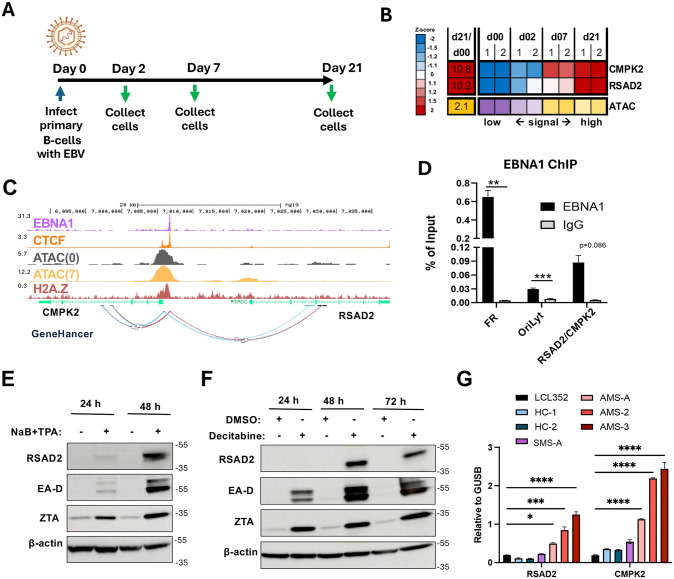
RSAD2 and CMPK2 are upregulated during EBV primary infection, lytic reactivation, and in Multiple Sclerosis (MS)-patient derived spontaneous lymphoblastoid cell lines (SLCLs) with unstable latency. **A**) Timeline of EBV infection (Mutu I derived) in primary B-cells. Virus icon Created in BioRender. Lieberman, P. (2026) https://BioRender.com/. rengv4z. **B**) RNA-seq and ATAC-seq signals after EBV infection of primary B-cells at day 0, 2, 7 and 21 days post-infection. Expression of RSAD2 and CMPK2 is shown as downregulated (blue) and upregulated (red) at different timepoints. Fold change in expression (left) at day 21 post-infection. **C**) Track representation with gene model and genehancer tracks in the context of EBNA1, CTCF, and H2A.Z ChIP-Seq and ATAC-Seq signal at day 0 and 7 post EBV infection. **D**) ChIP-qPCR assay for IgG control or EBNA1 at the EBV-FR (positive control), EBV oriLyt (negative control), or cellular CMPK2/RSAD2 promoter locus. **E-F**) Western blot analyses in Mutu I after treatment with sodium butyrate (NaB) +TPA for 24 or 48 hr (E) or Decitabine for 24, 48, or 72 hr **(F)**. Western blots were probed with antibody to RSAD2, EA-D, ZTA, or β-Actin. **G**) Expression analysis by RT-qPCR for RSAD2 (left) or CMPK2 (right) relative to GUSB in LCL352 or SLCLs derived from MS patients with active disease (AMS), stable disease (SMS) or healthy controls (HC). Statistical analysis was performed using ordinary two-way ANOVA using Sidak’s multiple comparisons (panels **D** and **G)**. n = 3, * p < .05, ** p < .01, ***p < .001, ****p < .0001.

A previous study from our group demonstrated that RSAD2/Viperin and CMPK2 are among the most upregulated genes following HIV infection of human macrophages, and that RSAD2/Viperin has a functional role in supporting sustained HIV infection of macrophages [15]. To investigate whether RSAD2/Viperin and CMPK2 represent a common response to chronic viral infections, we analyzed data from EBV infection of primary B-cells isolated from whole blood of normal healthy donors at various days after infection (Fig 1A) [16]. Analysis of the published RNAseq data revealed significant upregulation of RSAD2 and CMPK2 transcripts at days 2, 7 and 21 post-infection (Fig 1B). RSAD2 and CMPK2 genes share a common transcriptional regulatory region on chromosome 2 (Fig 1C). Examination of chromatin features of the common promoter/enhancer regulatory region of RSAD2 and CMPK2 revealed a strong activation of an ATAC-seq peak in response to EBV primary infection at day 7 (normalized peak value 12.2) compared to day 0 (normalized peak value 5.7) (Fig 1C). We also identified ChIP-seq peaks for EBNA1, CTCF, and H2A.Z that overlapped with the ATAC-seq peak suggesting that EBNA1 may directly regulate the chromatin accessibility and transcription of RSAD2 and CMPK2 (Fig 1C). To confirm that EBNA1 bound to the RSAD2/CMPK2 promoter/enhancer locus we performed ChIP-qPCR including positive control binding site at the EBV family of repeats (FR) and negative control region at the EBV OriLyt (Fig 1D). We found that EBNA1 bound to the RSAD2/CMPK2 locus with ~20% of the efficiency at viral FR element, but highly enriched relative to IgG control. Thus, EBV infection strongly induces expression of both RSAD2 and CMPK2 in primary B-cells through a mechanism involving EBNA1 binding and increased chromatin accessibility at the common promoter/enhancer regulatory region.

We next assayed RSAD2 and CMPK2 expression in B-cells latently infected with EBV. Western blot revealed that RSAD2 and CMPK2 proteins were undetectable in latently infected Mutu I cells but could be readily detected after treatment with lytic inducing agents NaB + TPA (Fig 1E) or Decitabine (Fig 1F) at 48 h post-induction, following expression of EBV lytic cycle early antigen EA-D and immediate early protein ZTA. We also found that RSAD2 along with Interferon-β (IFN-β) were induced in EBV^+^ Akata and LCL352, and to a lesser extent in EBV^-^ BJAB cells after treatment with NaB + TPA (S1 Fig). Additionally, expression of RSAD2 was more robust in Mutu I (EBV^+^) cells as compared to BJAB (EBV^-^) after treatment with NaB + TPA (S1C Fig). These findings suggest that reactivation of EBV occurring due to treatment with NaB + TPA in Mutu I leads to a more pronounced induction of RSAD2 in Mutu I, as compared to BJAB. Thus, EBV drives the induction of RSAD2 in Mutu I. These findings demonstrate that RSAD2 is upregulated during viral reactivation from latency in several different B-cell models of EBV latency, but may not be strictly dependent on EBV for its activation in B-cells.

We next tested whether RSAD2 and CMPK2 expression correlate with the intrinsic control of EBV latency. A previous study found that spontaneous lymphoblastoid cell lines (SLCLs) derived from MS patients had unstable latent infections with increased lytic cycle gene expression and inflammatory cytokine production compared to healthy controls [17]. We examined this cohort of SLCLs and found that patients with active MS (AMS) had significantly higher expression of both RSAD2 and CMPK2 as compared to LCL352 (Mutu I transformed) and SLCLs derived from healthy controls (HC) or stable disease (SMS) (Fig 1G). Expression levels of both genes were up to 10-fold higher in patients with active disease (Fig 1G) where lytic gene expression and inflammatory cytokines are elevated [17]. These findings suggest that RSAD2 and CMPK2 expression correlate with the EBV lytic activity and inflammatory state associated with active MS.

### Knockdown of RSAD2 modulates expression of EBV

**Fig 2:**
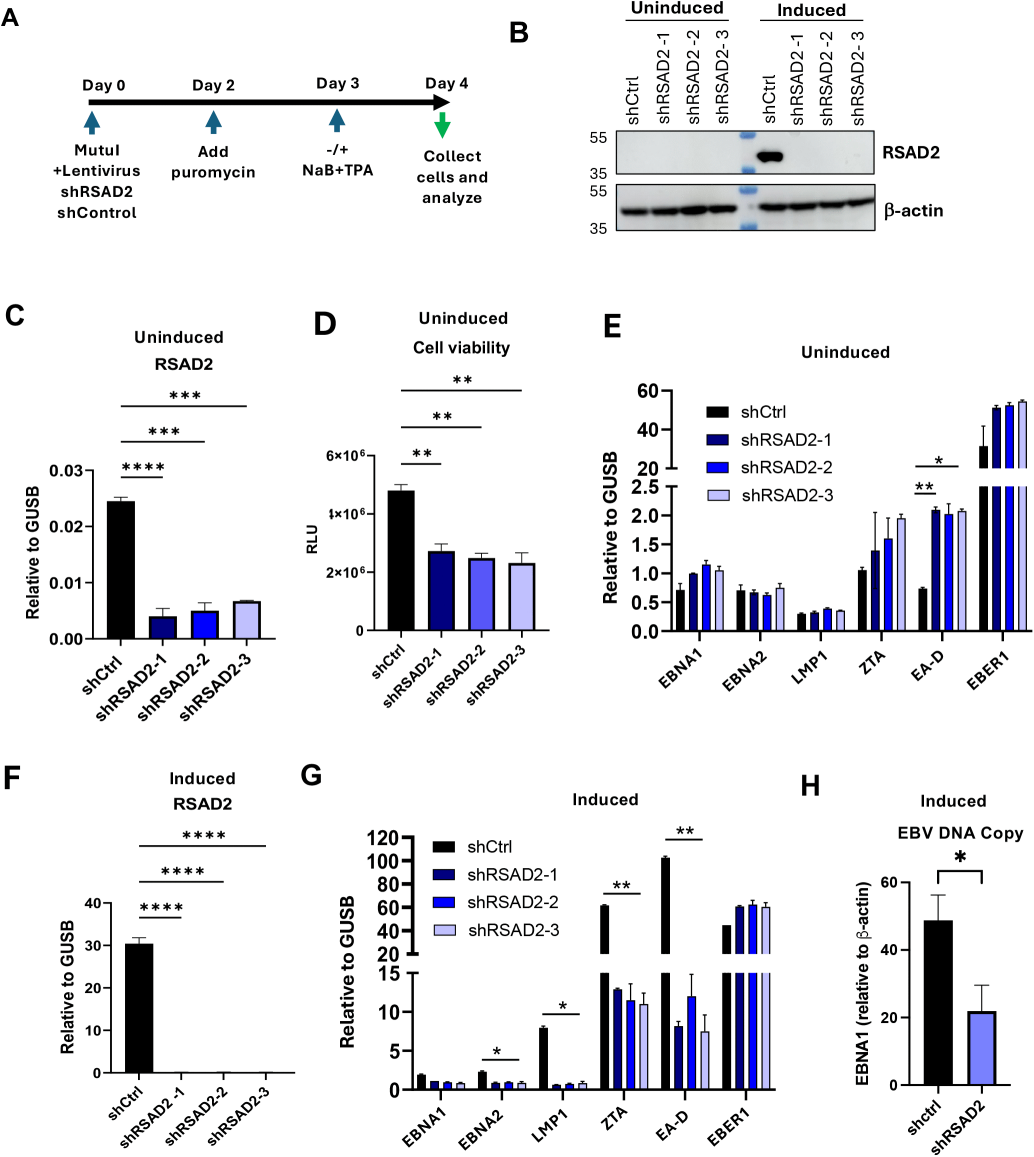
Knockdown of RSAD2 modulates EBV expression and cell viability. **A**) Schematic representation of timeline for RSAD2 knockdown by lentivirus transduction in Mutu I. **B**) Western blot for RSAD2 expression in Mutu I cells uninduced and induced (treated with NaB + TPA, 24 h) condition with shCtrl or three independent shRNAs for RSAD2. **C**) RT-qPCR analysis for RSAD2 expression after knockdown in uninduced Mutu I. **D**) Measurement of cell viability by CellTiterGlo assay in uninduced Mutu I after RSAD2 knockdown. **E**) EBV expression analysis by RT-qPCR in uninduced Mutu I. **F**) RT-qPCR analysis for RSAD2 expression after knockdown in induced Mutu I. **G**) RT-qPCR for EBV mRNA expression in induced Mutu I. **H)** EBV DNA copy number measured by qPCR for EBNA1 gene relative to β-actin. Statistical analysis was performed using ordinary one-way ANOVA using Dunnett’s multiple comparisons (panels **C**,**D** and **F**) or ordinary two-way ANOVA using Sidak’s multiple comparisons (panels **E** and **G)**. Data shown here is representative of results from three independent experiments. n = 3, * p < .05, ** p < .01, ***p < .001, ****p < .0001.

To investigate the effect of RSAD2 on EBV and cellular response during latency and lytic reactivation, we knocked down RSAD2 in Mutu I. We used three different shRNAs in either uninduced latency or after treatment with NaB + TPA to induce lytic EBV reactivation (Fig 2A). RSAD2 was efficiently knocked down by all three shRNAs as measured by Western blot in Mutu I cells induced by NaB + TPA (Fig 2B). More sensitive RT-qPCR analysis of RSAD2 transcript also demonstrated efficient knockdown of RSAD2 in uninduced Mutu I cells (Fig 2C). Knockdown of RSAD2 decreased cell viability relative to control shRNA by ~50%, suggesting that RSAD2 is essential for Mutu I cell survival (Fig 2D). RSAD2 knockdown in latently infected Mutu I cells had only a small effect on EBV transcripts, showing a modest increase in expression of EA-D (Fig 2E). Induction of EBV reactivation by treatment with NaB + TPA upregulated the expression of RSAD2 protein and transcript ~100-fold as compared to uninduced cells (Fig 2F compared to 2C), and shRNA depletion was highly efficient, with virtually no detectable RSAD2 protein (Fig 2B) or RNA (Fig 2F). While RSAD2 is thought to provide antiviral function, we found that knockdown of RSAD2 during lytic induction significantly reduced EBV lytic transcription, reducing expression of ZTA and EA-D by >80% (Fig 2G). Correspondingly, RSAD2 knockdown reduced EBV DNA copy number during lytic reactivation in NaB + TPA treated Mutu I cells as measured by qPCR (Fig 2H). These findings suggest that RSAD2 is required for maintaining B-cell survival during latency and efficient EBV transcription and DNA replication during EBV lytic induction.

### CMPK2 is antiviral and restricts EBV expression

**Fig 3:**
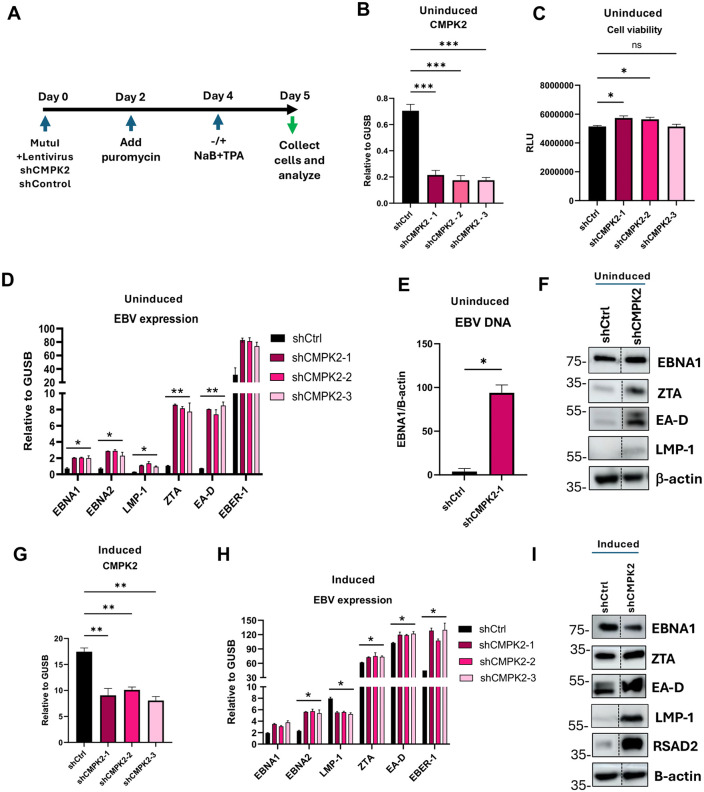
CMPK2 is antiviral and restricts EBV lytic expression. **A**) Schematic representation of timeline for CMPK2 knockdown in Mutu I using shCtrl and three independent shRNAs for CMPK2. **B**) RT-qPCR analysis for CMPK2 expression after knockdown in uninduced conditions. **C**) Measurement of cell viability by CellTiterGlo assay in uninduced conditions. **D**) EBV mRNA expression analysis by RT-qPCR in uninduced Mutu I. **E**) EBV DNA copy number measured by qPCR for EBNA1 gene relative to β-actin. **F**) Western blot analysis for EBV protein expression for uninduced Mutu I. Dashed line indicates cropping of lanes. **G**) RT-qPCR for CMPK2 expression after knockdown in induced conditions. **H**) EBV mRNA expression analysis by RT-qPCR for induced Mutu I. **I**) Western blot analysis for EBV protein expression under induced conditions. Dashed line indicates cropping of lanes. Statistical analysis was performed using ordinary one-way ANOVA using Dunnett’s multiple comparisons (panels **B, C** and **G)**,or ordinary two-way ANOVA using Sidak’s multiple comparisons (panels **D** and **H)**. Data shown here is representative of results from three independent experiments. n = 3, * p < .05, ** p < .01, ***p < .001, ****p < .0001.

To study the effect of CMPK2 on EBV expression and cellular response, we knocked down CMPK2 using three different shRNAs in Mutu I using experimental timeline as shown in Fig 3A. Under basal conditions, expression of CMPK2 is low and all three shRNAs efficiently knocked down CMPK2 (Fig 3B). Knockdown of CMPK2 did not have significant impact on cell survival under uninduced latency, although we observed a slight increase in cell viability (Fig 3C). Depletion of CMPK2 led to significant upregulation of EBV lytic gene expression in uninduced Mutu I cells as shown by RT-qPCR (Fig 3D), as well as a significant increase (~30 fold) in EBV DNA copy number indicating lytic replication was occurring (Fig 3E). Western blot also revealed an increase in lytic cycle protein ZTA and EA-D, as well as latency proteins EBNA1 and LMP1 (Fig 3F). Knockdown of CMPK2 during lytic EBV reactivation reduced CMPK2 mRNA transcripts by ~50% (Fig 3G), and led to minor changes to EBV transcripts, including increases in EBNA1, EBNA2, and EBER1, and decrease in LMP1 as detected by RT-qPCR (Fig 3H). Interestingly, Western blot showed an increase in EA-D and LMP1 protein, along with an increase in RSAD2/Viperin protein, consistent with the enhanced reactivation of EBV and potential translational control of LMP1 and RSAD2/Viperin during lytic activation (Fig 3I). These findings suggest that CMPK2 acts as a restriction factor for EBV during latent infection in Mutu I, and modulator of LMP1 and RSAD2/Viperin protein translation during lytic reactivation.

### Knockdown of RSAD2 leads to apoptosis while depletion of CMPK2 enhances cell survival

**Fig 4:**
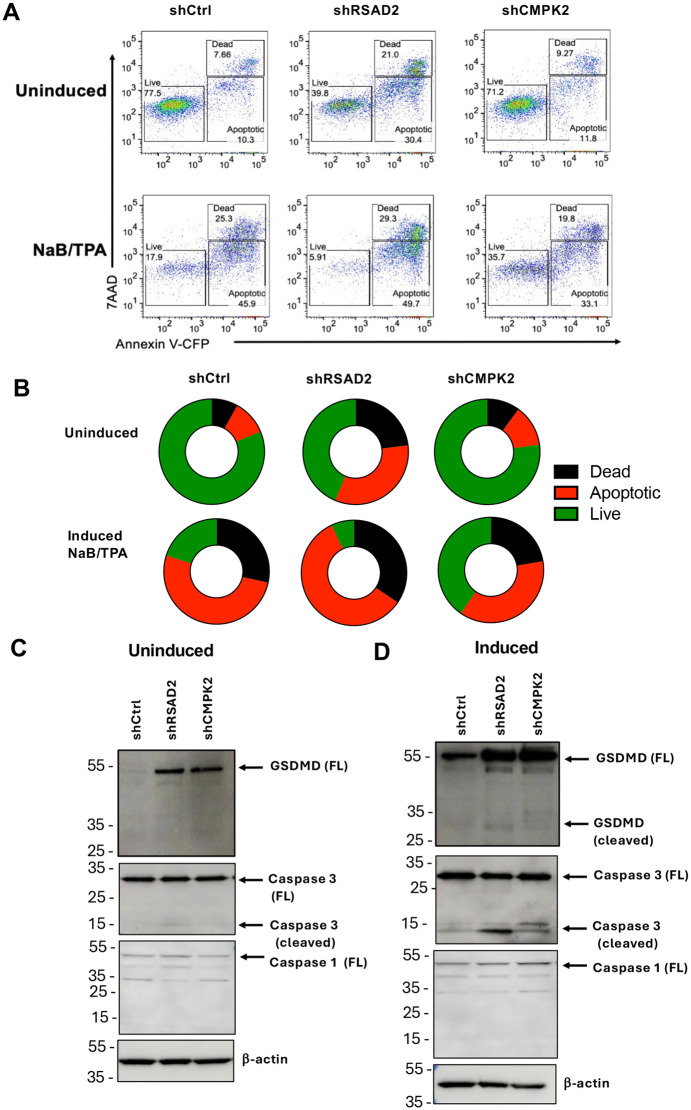
Knockdown of RSAD2 leads to apoptosis while depletion of CMPK2 enhances cell survival. **A**) Apoptosis assay by flow cytometry after Annexin V-7AAD staining in induced or uninduced Mutu I transduced with shCtrl, shRSAD2, or shCMPK2. **B**) Circle chart of live, dead, and apoptotic cells after treatment shown in panel A. **C-D**) Western blot analysis for expression of GSDMD, Caspase 1, Caspase 3, and loading control β-actin under uninduced condition **(C)**, and induced condition **(D)**.

Since we found that RSAD2 knockdown led to substantial cell death, we further wanted to study the effect of RSAD2 and CMPK2 knockdown on apoptosis. We analyzed for apoptosis by flow cytometry using Annexin V/7-AAD staining (Fig 4A). We found that loss of RSAD2 during uninduced and induced conditions lead to substantial cell death by apoptosis (Fig 4A and 4B). Under uninduced conditions, depletion of RSAD2 in Mutu I reduced cell viability from 78% to 39%, which accounts to ~50% decrease in live cell population (Fig 4A and 4B). Interestingly, while knockdown of CMPK2 under uninduced conditions had no significant effect on cell viability, depletion of CMPK2 during lytic EBV reactivation significantly enhanced cell viability (Fig 4A and 4B). Thus, CMPK2 may enhance EBV reactivation by preventing antiviral apoptotic response.

To better understand the role of RSAD2 and CMPK2 modulation on cell viability, we performed Western blot analyses. We found full-length Gasdermin D (GSDMD) was upregulated after RSAD2 and CMPK2 knockdown during uninduced (Fig 4C) and induced conditions (Fig 4D). However, we did not detect a cleaved form of GSDMD, which is a marker for pyroptosis [18]. Caspase 3 cleavage, a marker of apoptosis and pyroptosis [19], was detected after RSAD2 knockdown in uninduced and a more substantial cleavage signal in induced conditions (Fig 4C and 4D). Caspase 1 cleavage, a marker of inflammasome activity [20], was not detected under these conditions. Thus, a specialized mechanism of cell death, possibly involving early stage pyroptosis (elevated, but non-cleaved GSDMD), occurs after knockdown of RSAD2 in reactivating Mutu I cells.

### RSAD2 and CMPK2 are essential for induction of efficient IFN-response during lytic EBV reactivation

**Fig 5:**
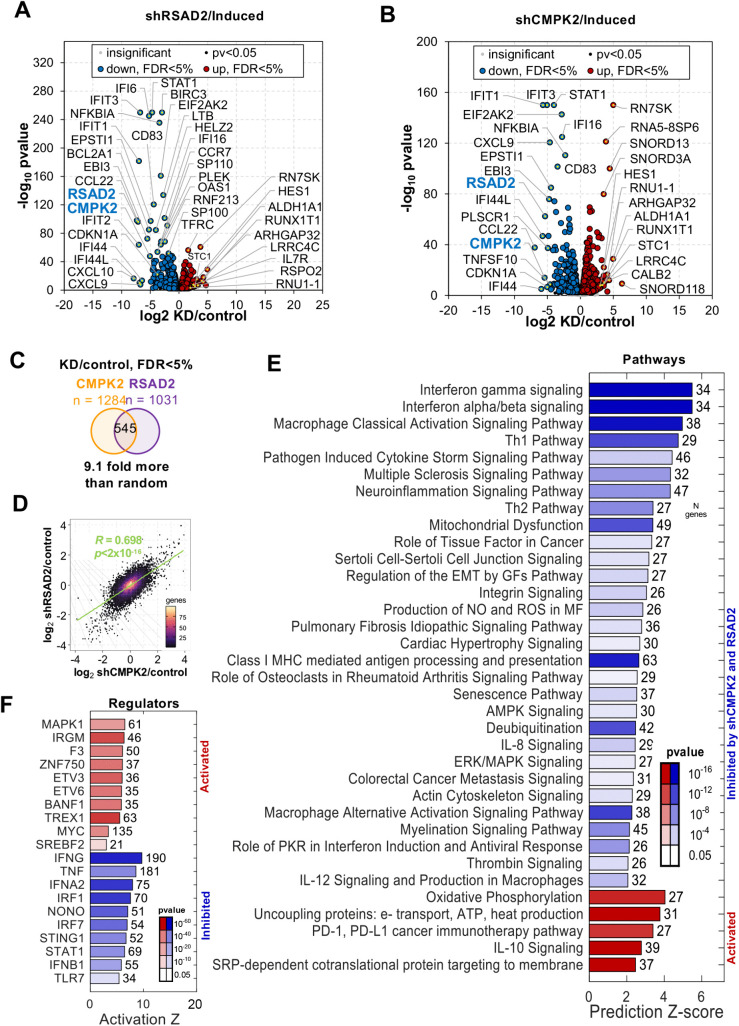
RSAD2 and CMPK2 are essential for induction of efficient IFN-response during lytic EBV reactivation. **A**) Volcano plot showing top significantly changed genes by shRSAD2 in Mutu I cells treated with sodium butyrate (NaB) and TPA to induce lytic EBV reactivation. **B**) Volcano plot showing top significantly changed genes by shCMPK2 in induced Mutu I cells. **C**) Venn diagram showing overlap of commonly regulated genes for shRSAD2 and shCMPK2. **D**) Pearson correlation of transcript changes in shCMPK2 and shRSAD2. **E**) Pathways analysis for downregulated (blue) and upregulated (red) genes common to both shCMPK2 and shRSAD2 in Mutu I cells. **F**) Regulators for down (blue) and up (red) activation based on Z-score using IPA.

To gain additional insight into the mechanism of RSAD2 and CMPK2 in regulating cellular response during latency and lytic EBV reactivation, we performed transcriptional profiling by RNA-seq after knockdown of either RSAD2 or CMPK2 (Figs 5 and S2-S4). In the absence of knockdown, NaB + TPA induction of Mutu I cells resulted in an upregulation of genes associated with IFN-α/β signaling, Cytokine Signaling, and downregulation of E2F Targets, G2M Checkpoints, and Myc Targets (S2 Fig). Knockdown of RSAD2 or CMPK2 had significant effects on both latent and lytic induced cells, but most strikingly were the similar effects on the reduction of Interferon signaling during lytic induction (Figs 5A-5E, S3 and S4). During lytic conditions, CXCL10, CCL22, IFIT2, IFI44L were most significantly downregulated by RSAD2 knockdown (Figs 5A, S4), while CCL22, IFI44, IFI44L, RSAD2, and IFIT3 were most significantly downregulated by CMPK2 knockdown (Figs 5B, S4). Knockdown of either RSAD2 or CMPK2 during lytic induction strongly induced expression of ARGHAP32 and LRRC4C, which are implicated in neuronal development and function. We found a highly significant overlap (Fig 5C) and correlation (Fig 5D) in genes regulated by RSAD2 or CMPK2 knockdown under induced conditions. Common pathways most significantly down regulated were IFN gamma signaling, IFN alpha/beta signaling, Macrophage Th1 activation pathway, Mitochondrial dysfunction, Antigen presentation and Deubiquitination (Fig 5E). Common pathways most upregulated were Oxidative Phosphorylation, IL-10 signaling, Protein translation, PD-1/PD-L1 immunotherapy pathway and SRP-dependent cotranslational targeting (Fig 5E). Regulators most affected were inhibition of IFN-γ, TNF, IFN-α2 and activation of MAPK1 and immunity related GTPase IRGM (Fig 5F). Top downregulated genes following knockdown of both, RSAD2 and CMPK2 under uninduced latency were SMIM15, CCL22 and GBP1 (S2B and S2C Fig). We experimentally validated the most affected common genes CXCL10, CCL22, IFI-6 were reduced by shRSAD2 (S5A Fig) and shCMPK2 (S5B Fig), while RUNX1T1 was upregulated by both RSAD2 and CMPK2 knockdown. Thus, knockdown of RSAD2 and CMPK2 have a highly similar effect on the host transcriptional response to EBV latent state and lytic reactivation.

### CMPK2 localizes to mitochondria and affects mitochondrial transcription

**Fig 6:**
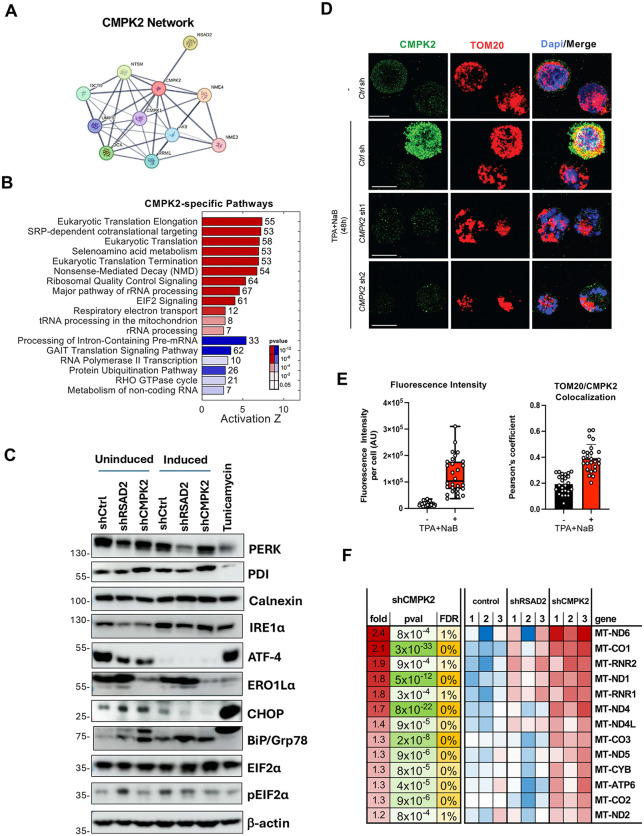
Knockdown of CMPK2 increases mitochondrial transcription and alters ER homeostasis. **A**) STRING database interaction map for CMPK2. **B**) Pathway analysis of RNA-seq for genes uniquely affected by CMPK2 knockdown as activated (red) or inhibited (blue) during lytic EBV reactivation induced by treatment with sodium butyrate (NaB) and TPA in Mutu I. **C**) Western blot for shCtrl, shRSAD2, or shCMPK2 in uninduced or induced Mutu I cells probed with antibodies to ER stress markers PERK, PDI, Calnexin, IRE1α, ATF-4, ERO1Lα, CHOP, BiP/Grp78, EIF2α, and control β-actin. **D**) Representative confocal images for CMPK2 and mitochondrial marker Tom20 colocalization in Mutu I cells with/without NaB + TPA treatment and either shCtrl or shCMPK2 transduction. DAPI, nuclei (blue). Scale bars,10 μm. **E)** Quantification of fluorescence intensity of CMPK2 (left; *n* = 28 cells per group) and co-localization of CMPK2 with TOM20 (right; *n* = 25 cells per group). **F)** Transcriptomic analysis of mitochondrial gene expression by RNA-seq after RSAD2 and CMPK2 knockdown.

While RSAD2 and CMPK2 have overlapping effects on IFN signaling, mitochondrial dysfunction and antigen presentation, the two proteins are thought to have different sub-cellular localization and additional gene-specific effects. We first examined the pathways specific for CMPK2 (Fig 6). Analysis of gene interactions from the STRING database (https://string-db.org) identified genes involved in nucleotide metabolism, including RSAD2, NT4M, AK9, and RRM1 (Fig 6A). Analysis of RNA-seq data during induced conditions identified pathways specific to CMPK2-knockdown to be Eukaryotic Translation, Ribosome Quality Control, Respiratory Electron Transport as activated, while Protein Ubiquitination was inactivated (Fig 6B). We therefore assayed the effects CMPK2 (as well as RSAD2) knockdown on ER stress and Unfolded Protein Response (UPR) regulatory factors by Western blot analysis (Fig 6C). We found that CHOP and BiP/Grp78, both of which are regulators of UPR, localized in the ER, were upregulated after CMPK2 and RSAD2 knockdown in uninduced conditions. Levels of Protein Disulfide Isomerase (PDI) slightly increased after CMPK2 knockdown while ERO1Lα levels significantly reduced under both uninduced and induced states. ATF4 levels decreased substantially after lytic induction, and knockdown of either CMPK2 and RSAD2 reduced ATF4 in uninduced conditions. peIF2α levels were slightly elevated after RSAD2 knockdown in uninduced conditions, further suggesting an effect on protein translation (Fig 6C). Thus, knockdown of both CMPK2 or RSAD2 alter UPR and ER homeostasis. To gain further insight into CMPK2 function, we analyzed its subcellular localization by confocal imaging in Mutu I cells. As expected, CMPK2 localized primarily to mitochondria inner matrix as indicated by partial colocalization with TOM20, a known biomarker for the outer mitochondrial membrane [21] (Fig 6D and 6E). EBV lytic induction in Mutu I cells resulted in a strong upregulation of CMPK2 at mitochondria, and this signal was reduced by shCMPK2 along with some changes in TOM20 signal, suggestive of mitochondrial dysfunction (Fig 6D and 6E). Consistent with a role in mitochondrial function, we observed shCMPK2, and to a lesser extent shRAD2, resulted in an increase in transcription of several mitochondrial genes, including MT-ND6, MT-CO1 and MT-RNR2 (Fig 6F), further implicating its role in maintaining mitochondrial homeostasis.

### RSAD2 localizes to the ER and affects TRAF6-IRAK1 protein levels and LMP1-dependent NF-κB signaling

**Fig 7:**
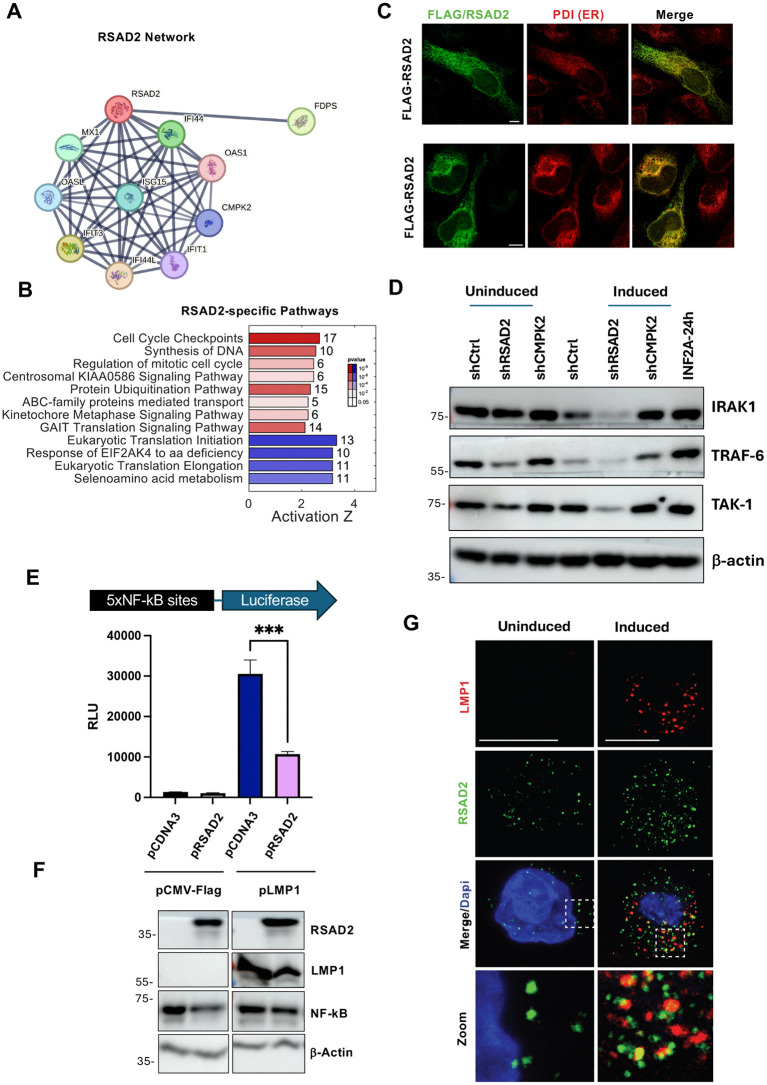
RSAD2 localizes to the ER and affects TRAF6-IRAK1 protein levels and LMP1-dependent NF-kB signaling. **A**) STRING database interaction map for RSAD2. **B**) Pathway analysis of RNA-seq for genes uniquely affected by RSAD2 knockdown as activated (red) or inhibited (blue) during lytic EBV reactivation induced by treatment with sodium butyrate (NaB) and TPA in Mutu I. **C**) Representative confocal micrographs of HeLa cells transfected with FLAG-RSAD2 (FLAG or RSAD2, green), ER marker PDI (red), and merge. Scale bars, 10 µm. **D**) Western blot analysis for expression of TLR/NF-kB signaling proteins IRAK1, TRAF-6, and TAK-1 in uninduced or induced Mutu I cells treated with shCtrl, shRSAD2, or shCMPK2. **E**) Luciferase assay for 5xNF-kB-Luciferase plasmid co-transfected with either pcDNA3.1, pRSAD2, pCMV-FLAG, or pLMP1 (as indicated) in 293T cells. **F**) Western blot showing expression of RSAD2, LMP1, NF-kB, and β-Actin in 293T cell luciferase assays shown in panel E. **G**) Representative confocal images of LMP1 (red), RSAD2 (green) in Mutu I cells uninduced or induced with NaB + TPA for 48 hrs. Inset: LMP1/RSAD2 colocalization. DAPI, nuclei (blue). Scale bars, 10 μm.

We next examined pathways more specific for RSAD2 (Fig 7). STRING database analysis for RSAD2 showed an interaction with CMPK2, but most interactions were with other IFN-stimulated genes, such as IFI44, OAS1 and MX1 (Fig 7A). Pathway analysis for RNA-seq specific to RSAD2-knockdown identified Cell Cycle Checkpoints and Protein Ubiquitination as activated, while Eukaryotic Translation pathways were inactivated (Fig 7B). Thus, while CMPK2 and RSAD2 affect common pathways, some of these effects were in opposing directions. Ectopic expression of FLAG-tagged RSAD2 in 293T cells revealed a subcellular localization with endoplasmic reticulum using PDI as a marker (Fig 7C). This localization is consistent with RSAD2 affecting Eukaryotic Translation, and the UPR (Fig 7B). Given the common role of both RSAD2 and CMPK2 in TLR signaling, we next examined the effects of RSAD2 and CMPK2 knockdown on TLR signaling molecules that have also been implicated as RSAD2 interaction partners, such as IRAK1 and TRAF6 [22]. We found that RSAD2 knockdown reduced expression levels of IRAK1, TRAF6 and TAK1 relative to β-actin during induced, and to a lesser extent, uninduced conditions (Fig 7D). Knockdown of CMPK2 increased expression of IRAK1 and TRAF6 under induced conditions as compared to shCtrl (Fig 7D). Thus, depletion of CMPK2 may modulate TLR signaling via IRAK1 and TRAF6. Because TRAF6 is known to interact with EBV latency membrane protein 1 (LMP1) and regulate NK-κB activation [23], we assayed the effects of RSAD2 on an NF-κB-responsive reporter gene (NF-κB-Luciferase) in HeLa cells co-transfected with expression vectors for RSAD2 alone or in combination with LMP1 (Fig 7E and 7F). As expected, LMP1 strongly activated NF-κB-Luciferase. Interestingly, co-expression of RSAD2 reduced NF-κB activation by LMP1 ~ 3 fold (Fig 7E). We also observed that RSAD2 partially colocalized with LMP1 in Mutu I cells when induced by NaB + TPA (Fig 7G). Taken together, these findings suggest that RSAD2 regulates NF-κB signaling mediated by LMP1 potentially through regulation of TRAF6 and IRAK1.

### RSAD2-dependent generation of ddhCTP and global metabolite shift during EBV reactivation

**Fig 8:**
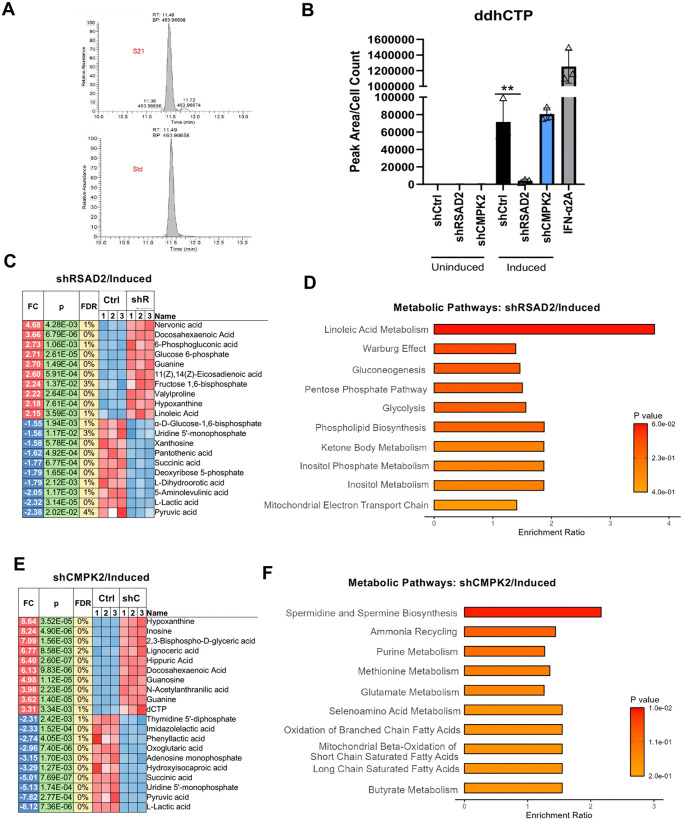
ddhCTP detection after RSAD2 and CMPK2 knockdown and global metabolite shift during EBV reactivation. **A)** LC-MS chromatogram of ddhCTP standard and its detection in the sample. **B)** Detection of ddhCTP in control (shCtrl) RSAD2 knockdown (shRSAD2) and CMPK2 knockdown (shCMPK2) in Mutu I cells under uninduced and induced condition. **C)** Top 10 up/downregulated metabolites after RSAD2 knockdown in induced Mutu I cells. **D)** Pathway enrichment analysis for RSAD2 knockdown in induced Mutu I cells. **E)** Top 10 up/downregulated metabolites after CMPK2 knockdown in induced Mutu I cells. **F)** Pathway enrichment analysis for CMPK2 knockdown in induced Mutu I cells.

To investigate the role of RSAD2 and CMPK2 on metabolic response to EBV, we examined the effects of these enzymes on cellular metabolites. RSAD2 is known to catalyze the conversion of CTP into ddhCTP, a unique antiviral ribonucleotide that can act as a chain terminator for viral encoded RNA-dependent RNA polymerases [6]. CMPK2 functions to provide CTP substrate for conversion to ddhCTP by RSAD2 [6]. To determine if ddhCTP was produced during lytic EBV reactivation and if knockdown of RSAD2 and CMPK2 affected ddhCTP formation, we used LC-MS to measure ddhCTP levels (Fig 8A and 8B). LC-MS identification was calibrated using purified synthetic ddhCTP (Fig 8A). Under basal conditions (uninduced), ddhCTP was nearly undetectable (Fig 8B). However, during lytic EBV reactivation, ddhCTP levels were substantially increased in control cells (shCtrl, induced) likely due to increased expression of both RSAD2 and CMPK2 (Fig 8B). Knockdown of RSAD2 during lytic induction led to almost complete loss of ddhCTP, showing that RSAD2 is indispensable for ddhCTP production (shRSAD2, induced). In contrast, knockdown of CMPK2 did not significantly decrease ddhCTP. IFN-α2A treated cells showed very high levels of ddhCTP formation, as expected. We also found that EBV reactivation induced cellular levels of ddhCMP (S6A Fig), and ddhCDP (S6B Fig). Interestingly, knockdown of RSAD2 reduced ddhCDP levels (S6B Fig), but not ddhCMP (S6A Fig), suggesting alternative pathways may generate ddhCMP. These findings show the strong correlation between EBV reactivation and the RSAD2 dependent generation of ddhCTP and its related byproducts.

To gain a broader understanding of metabolite changes during EBV reactivation and in response to RSAD2 or CMPK2 knockdown, we used mass-spectrometry for global metabolite profiling. Many metabolic changes were observed for each condition (Figs 8C-8F, S7). Knockdown of RSAD2 significantly increased levels of Nervonic acid, Docosahexaenoic acid, Linoleic acid, Glucose 6-phosphate, and Fructose 1,6-bisphosphate (Fig 8C and 8D), while knockdown of CMPK2 increased levels of metabolites such as Hypoxanthine, Inosine, Lignoceric acid, Docosahexaenoic acid and 2,3-bisphospho glyceric acid (Fig 8E and 8F). Metabolite changes occurring between uninduced and induced conditions also showed similarities. For example, under uninduced state knockdown of RSAD2 increased levels of Fructose 1,6-bisphosphate, Nervonic acid, and Lignoceric acid (Figs 8 and S7A). Metabolites such as Nervonic acid, Lignoceric acid, Docosahexaenoic acid are involved in neurodevelopment, while Glucose 6-phosphate, and Fructose 1,6-bisphosphate are intermediates in glycolysis. Nervonic acid helps in remyelination of nerves in Multiple Sclerosis and also has anti-inflammatory properties. In terms of pathway enrichment, Spermidine and Spermine Biosynthesis had high enrichment ratio for CMPK2 knockdown and, Alphalinoleic acid and Linoleic acid metabolism was enriched in RSAD2 knockdown. There was a significant overlap in the metabolites regulated by both genes including decreased levels of Pyruvic acid, Lactic acid and Succinic acid. We also tested whether IFN treatment altered global metabolites (S7E and S7F Fig). Metabolites that changed most with IFN-α treatment included Sepiapterin, Uridine, Cytidine, Lignoceric acid, Docosahexanoic acid, dCMP, dCTP, Lactic acid, Pyruvic acid and Succinic acid. Thus, the metabolic profile overlapped to some extent between IFN-α-treated cells and knockdown of either RSAD2 or CMPK2, suggesting these enzymes affect common metabolic pathways. These findings demonstrate the RSAD2 and CMPK2 play important roles in regulating cellular metabolism during EBV latency and reactivation in B-lymphocytes.

## Discussion

**Fig 9:**
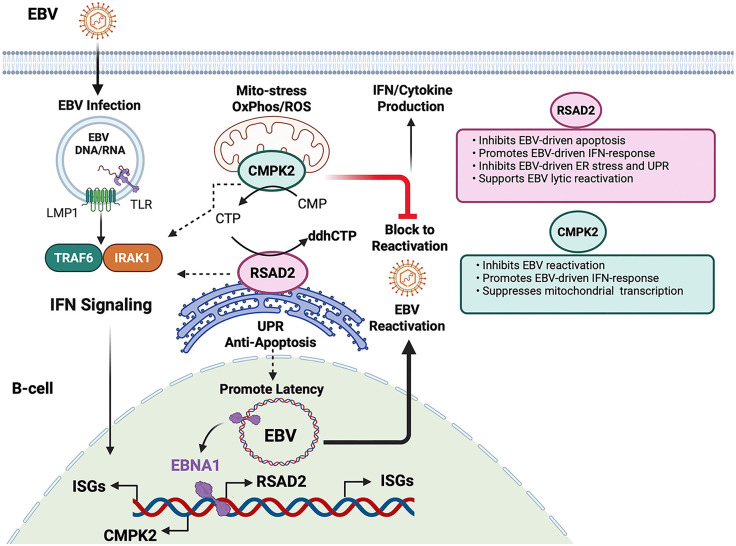
Model showing the interrelated role of RSAD2 and CMPK2 in regulating immune-metabolic response to EBV latency and reactivation. Created in BioRender. Lieberman, P. (2026) https://BioRender.com/sfkebcy.

In the present study, we identified RSAD2/Viperin and CMPK2 as host genes induced by EBV infection and demonstrate their functional roles in modulating host response to viral latency and reactivation. The common transcriptional control region of RSAD2 and CMPK2 was bound by EBNA1 and its chromatin accessibility increased by EBV infection. shRNA depletion of RSAD2 and CMPK2 revealed their common function in activation of IFN response genes during EBV reactivation in B-lymphocytes. RSAD2 depletion activated host cell apoptosis pathways, including cleavage of Caspase 3, and loss of IFN signaling during EBV reactivation. CMPK2 depletion led to a spontaneous reactivation of latent EBV, suggesting it functions as an EBV lytic cycle restriction factor. While RSAD2 is localized to the ER and CMPK2 localized to the mitochondria, these IFN-inducible factors function coordinately during EBV infection and reactivation to modulate host UPR, mitochondrial function, metabolic pathways, NF-κB signaling and IFN-response that promote infected cell survival and viral latency (Fig 9).

RSAD2 and CMPK2 are coordinately regulated during EBV infection and lytic reactivation of B-cells. We found that EBNA1 can bind to the common promoter region regulating the divergently transcribed genes, and that ATAC-seq chromatin accessibility increased during EBV infection. EBNA1 binding site overlapped with histone H2A.Z and in close proximity to CTCF suggesting complex and higher order chromatin regulation. RSAD2 and CMPK2 are known ISGs that can be induced directly by IFN signaling. Thus, EBV is likely to directly modulate expression of RSAD2 and CMPK2 through EBNA1 and other viral factors.

Multiple studies have demonstrated that RSAD2/Viperin is highly upregulated following viral infection and can restrict viral replication [5,6,24]. However, other studies, particularly with DNA viruses, have found a proviral function for RSAD2/Viperin. RSAD2/Viperin was found to support Kaposi’s sarcoma-associated herpesvirus lytic replication through methionine oxidation and stabilization of the viral helicase [25]. During HCMV infection, RSAD2/Viperin is relocalized to mitochondria where it inhibits ATP production, activating AMPK, and triggering lipogenesis to support enveloped virion assembly [26]. In a previous study conducted by our group in HIV-infected macrophages, we found reduction in HIV transcripts and p24 viral capsid protein levels after RSAD2 knockdown, showing that RSAD2 is essential for sustained HIV infection of human macrophages [27]. In our present study, we found a reduction in EBV lytic transcription after RSAD2 knockdown, partly due to the increase in apoptosis and activation of UPR. Thus, RSAD2 provides a proviral function in maintaining cell viability and promoting EBV lytic gene expression. Thus, RSAD2 can serve as a proviral survival factor for EBV, as well as for other DNA viruses.

RSAD2 and CMPK2 knockdown had opposing effects on viral reactivation and cell viability. RSAD2 depletion reduced EBV lytic DNA replication and triggered substantial cell death (Figs 2 and 4). RSAD2 knockdown increased Annexin V/7AAD- positive cells and the accumulation of cleaved Caspase 3, indicating apoptosis [18]. We also observed an increase in full-length GSDMD, but did not detect the cleaved form associated with pyroptosis [28,29]. Thus, precise mechanism of apoptosis remains to be determined. In contrast, CMPK2 knockdown increases cell viability during lytic reactivation and led to spontaneous reactivation in latently infected cells. Thus, CMPK2 can be considered a restriction factor for EBV latency.

CMPK2 knockdown also led to a reciprocal increase of RSAD2/Viperin protein during EBV lytic reactivation (Fig 3H). The increase in RSAD2/Viperin protein level after CMPK2 knockdown was not reflected in RSAD2 transcript levels and therefore could reflect post-transcriptional regulation. This would be consistent with the role of CMPK2 in translational control mechanisms, as indicated by RNA-seq (Fig 6). Increased RSAD2/Viperin protein after CMPK2 knockdown may also be a result of EBV reactivation. Since RSAD2/Viperin is an important survival factor, it is possible that high RSAD2/Viperin protein accumulation after CMPK2 knockdown may be increasing cell survival.

RSAD2/Viperin is ER/lipid droplet-associated [30], and CMPK2 is mitochondrial [31], positioning them at opposite ends of an ER–mitochondria stress axis. Viperin’s N-terminal amphipathic helix targets the ER/lipid droplets, domains essential for membrane biogenesis and secretory load, suggesting that Viperin may support ER homeostasis under infection induced proteostatic stress [32]. Increased BiP/GRP78 and CHOP, peIF2α elevation, and ATF4 reduction after RSAD2 or CMPK2 knockdown indicate UPR remodeling (Fig 6C). Although the canonical PERK-eIF2α-ATF4-CHOP cascade often elevates ATF4, contexts exist where sustained eIF2α phosphorylation enforces global translation attenuation with disparate ATF4 dynamics, particularly under combined hypoxia/oxidative stress, pointing to non-canonical UPR tuning during EBV reactivation. Notably, EBV encoded oncoprotein LMP1 was found to inhibit the integrated stress response (ISR) by reducing expression of ATF4 and CHOP, and reducing phosphorylation of PERK and GCN2 in viral infected epithelial cells [33]. Additionally, UPR effectors, such as the spliced transcription factor XBP1, directly activates BZLF1, and UPR-inflammasome crosstalk can unlock lytic entry in B-cell lymphomas, providing one route by which organelle stress intersects EBV reactivation [34,35].

Our transcriptomic analysis revealed a critical role for RSAD2 and CMPK2 in regulating IFN response. EBV reactivation induced a strong IFN response that was severely suppressed by knockdown of either RSAD2 or CMPK2. This attenuation of immune signaling underscores the importance of RSAD2 and CMPK2 in maintaining antiviral defense mechanisms. Interestingly, despite the distinct enzymatic functions of RSAD2 and CMPK2, their knockdowns resulted in highly overlapping transcriptional profiles, suggesting a coordinated regulatory axis (Fig 5E and 5F). Both genes are highly evolutionarily conserved, occur immediately adjacent to each other and are co-expressed during IFN signaling. This shows conserved functional cooperation. Both genes modulated a shared set of pathways, including IFN-response, MAPK1 signaling, oxidative phosphorylation, electron transport, ATP production, cholesterol biosynthesis, SRP-dependent cotranslational protein targeting to membrane as well as immune-related pathways such as PD1-PD-L1 cancer immunotherapy signaling. The consistent downregulation of genes such as STAT1, and DDX58 (RIG-I) supports the notion that RSAD2 and CMPK2 are upstream regulators of the IFN signaling cascade. We also observed an increase in Interleukin-1 family signaling after RSAD2 knockdown. These findings point to a dual role for RSAD2 and CMPK2 in immunometabolism, linking antiviral defense to mitochondrial and metabolic reprogramming.

RSAD2 and CMPK2 are enzymes that function together in the generation of the antiviral metabolite ddhCTP. We found that ddhCTP levels were highly elevated by EBV reactivation, and that this was strictly dependent on RSAD2 (Fig 8B). RSAD2 uses CTP as the substrate to generate ddhCTP, and CTP can be generated by CMPK2, suggesting a direct metabolic handoff from CMPK2 to RSAD2. Interestingly, knockdown of CMPK2 did not reduce ddhCTP levels, suggesting alternative sources of CTP substrate are available for RSAD2. ddhCTP may also be generated from ddhC by other kinases such as UCK2, CMPK1 and NDP to generate ddhCTP [36]. A recent study identified ddhC, as an acute phase reactant in serum of individuals infected with SARS-CoV2 and Influenza A virus [37]. Another study implicated RSAD2 generation of ddhCTP as an inhibitor of NAD-dependent enzymes in the TCA cycle [38], although this finding remains controversial [36]. RSAD2 was also implicated in control of mitochondrial transcription through inhibition of mitochondrial RNA polymerase by ddhCTP [39]. Consistent with this observation, we found that CMPK2 and RSAD2 knockdown increased mitochondrial transcripts (Fig 6F), suggesting a potential mechanism for ddhCTP generation in regulating mitochondrial function and inflammatory signaling that would impact EBV latency and reactivation.

In addition to ddhCTP, global metabolite profiling revealed many other changes in response to EBV reactivation and knockdown of either CMPK2 or RSAD2. Depletion of CMPK2 led to an increase in Inosine/Hypoxanthine, a hallmark of purine salvage/catabolism flux which often rises with redox/nucleotide stress [40]. Pathway enrichment for polyamine biosynthesis, Alpha linolenic/linoleic acid metabolism, superoxide degradation and glycolysis all point towards mitochondrial nucleotide stress and redox changes (mtROS). The rise in nervonic (24:1), lignoceric (24:0), and DHA (22:6) implies elongase/desaturase activity involved in polyunsaturated fatty acid (PUFA) elongation and membrane domain architecture that viruses and IFN programs often modulate. Our data shows a coordinated RSAD2/CMPK2 program that couples mitochondrial nucleotide metabolism and redox to glycolysis and lipid elongation, and IFN-α superimposes a similar metabolic signature (particularly nucleotide and lipid remodeling), indicating the ISG axis is a major driver of these metabolic states.

Consistent with this immune inflammatory theme, our study revealed significant upregulation of both RSAD2 and CMPK2 in SLCLs derived from patients with active MS. These findings support a model in which both genes amplify IFN-driven inflammation while influencing EBV dynamics, potentially exacerbating MS pathogenesis.

Collectively, our study highlights RSAD2 and CMPK2 as central regulators of innate immune reprogramming during EBV infection and reactivation. Their influence extends beyond canonical IFN signaling to encompass mitochondrial function, ribosome biogenesis, ER-associated unfolded protein response and antiviral metabolites. The interplay between these pathways may be critical for balancing immune activation with cellular homeostasis, and further investigation into their roles could uncover novel therapeutic targets for EBV-associated diseases and autoimmune conditions.

## Materials and methods

### Human PBMCs, spontaneous lymphoblastoid cell lines and ethics statement

Spontaneous lymphoblastoid cell lines (SLCLs) and peripheral blood mononuclear cells (PBMCs) from MS patients and controls were obtained from collaborators at the National Institutes of Health (NINDS), University of Pennsylvania, Perelman School of Medicine and the Wistar Institute, as described previously [17]. All samples were de-identified and obtained with signed informed consent from participants according to IRB approval process at NIH (Protocol No. 89N0045 and CR0045), University of Pennsylvania (Protocol No. 816805, IRB Protocol No. 4) and the Wistar Institute (Protocol No. 22010335*)* collaboration agreement.

### Cells and treatments

Mutu I, LCL352, BJAB, Akata and SLCLs from MS patients were cultured in RPMI supplemented with 10% FBS, 2 mM L-glutamine, 100 U/ml Penicillin and 100 µg/ml Streptomycin. For inducing lytic EBV reactivation, cells were treated with 2 mM sodium butyrate (NaB) and 20 ng/ml TPA (12-O-tetradecanoylphorbol-13-acetate) for 24 h. Mutu I cells were treated with Decitabine, a global hypomethylating agent at final concentration of 7.5 µM for 24, 48 and 72 h. For IFN-α treatment, cells were treated with 10 ng/ml IFN-α2A (STEMCELL Technologies, 78076) for 24 h. For all treatments, either untreated cells or cells treated with DMSO were included as negative control.

### shRNA knockdown of RSAD2 and CMPK2

Lentiviruses were generated by cotransfecting pLKO.1 based shRNA expression plasmids obtained from The Wistar Institute Molecular Screening Facility with plasmids pMD2.G and pSPAX2 in 293T cells using Lipofectamine 2000 transfection reagent (ThermoFisher Scientific). Three days after transfection, supernatant containing the lentivirus was collected, centrifuged to remove cell debris, filtered through a 0.45 micron filter, aliquoted and stored at -80 °C until use. For gene knockdown, cells were resuspended in the lentivirus along with polybrene at a final concentration of 8 µg/ml followed by spinoculation at 450 g at room temperature for 1.5 h. After spinoculation, lentivirus was removed and cells were resuspended in fresh medium. After 48 h, puromycin was added to the culture medium at a final concentration of 2 µg/ml. Fresh medium containing puromycin was added to cells every 2–3 days until collection. For inducing lytic EBV reactivation in cells after knockdown, cells were treated with NaB + TPA for 24 h as described previously. These cells will be referred to as “Induced” hereafter. Cells not treated with NaB + TPA (uninduced) were also cultured alongside induced cells.

### Gene expression analysis by RT-qPCR

Cells were collected after treatments and washed once with PBS. RNA was isolated from cells using RNeasy plus mini kit. RNA was treated with DNase I (Roche, 4716728001) at room temperature for 20 min to remove residual genomic DNA. RNA was reverse transcribed to generate cDNA using High-capacity cDNA reverse transcription kit (ThermoFisher, 4368814). RT-qPCR was performed using SYBR green mastermix (ThermoFisher) in a Quantstudio 7 qPCR machine (Applied Biosystems), and ΔCt method was used for relative quantitation. No-template controls and no-reverse transcriptase controls were included in all RT-qPCR reactions. Data were normalized to the housekeeping gene GUSB. Primer sequences for RT-qPCR are listed in S1 Table.

### Cell viability

Cell viability was determined by CellTiter-Glo assay (Promega, G7570) as per manufacturer’s instructions. Briefly, cells were lysed using lysis reagent and relative luminescence units (RLU) were measured using CLARIOstar Plus microplate reader (BMG Labtech).

### Western Blot

Cell lysates were prepared in lysis buffer containing protease and phosphatase inhibitor cocktail (ThermoFisher, 78440). Protein concentration was determined using bicinchoninic acid (BCA) protein assay (Pierce) and lysates were subsequently boiled with 4X Laemmli sample buffer (Bio-Rad) containing β-mercaptoethanol. Proteins were analyzed by SDS-polyacrylamide gel electrophoresis (PAGE) on an 8–16% Tris-glycine precast gel (Invitrogen) and transferred to an Immobilon-P membrane (Millipore). Membranes were blocked in Tris-Buffered Saline containing 5% milk and 0.1% Tween-20, followed by incubation with primary antibody against RSAD2 (Cell Signaling Technology, 13996), CMPK2 (Abcam, 194567), EBNA1 (in-house generated antibody in rabbit), ZTA (in-house generated in rabbit), LMP-1 (Millipore Sigma, MABF2248), EA-D (Millipore Sigma, MAB8186), IRAK1 (Cell Signaling Technology), TRAF6 (Cell Signaling Technology, 8028S), TAK1 (Cell Signaling Technology, 5206), NF-κB (Cell Signaling Technology, 8242T), GSDMD (Cell Signaling Technology, 36425T), Caspase 3 (Cell Signaling Technology, 14220), Caspase 1 (Cell Signaling Technology, 3866), eIF2α (Cell Signaling Technology 5324T), p-eIF2α (Cell Signaling Technology 3398S), ATF-4 (Cell Signaling Technology, 11815T) or β-actin (Sigma-Aldrich, A3854). Proteins expressed during ER stress were detected using ER Stress antibody sampler kit (Cell Signaling Technology, 9956T). Membranes were washed with TBST, incubated for 1 h with the goat anti-rabbit IgG-HRP (Biorad, 1706515) or goat anti-mouse IgG-HRP (Biorad, 1706516). Membranes were then washed and detected by enhanced chemiluminescence using Amersham Imager 680 (GE Healthcare).

### Flow cytometry

For flow cytometry analysis, cells were first gated on FSC-A vs. SSC-A to define the primary cell population followed by singlets identification using FSC-A vs. FSC-H (Fig 4A). All downstream analysis was performed on resulting cell population. Cells were stained using Annexin V-7AAD Apoptosis Detection Kit (ThermoFisher Scientific) as per manufacturer’s instructions and analyzed by FACSymphony A5 SE flow cytometer (BD Biosciences). Data analysis was performed using FlowJo software v10.10.0 (BD Biosciences).

### EBV DNA quantification

Cells were harvested by centrifugation and washed once with PBS. DNA was extracted from cells using DNeasy Blood and Tissue kit (Qiagen, 69504) as per manufacturer’s instructions. DNA concentration was determined using Qubit fluorometer. Quantitative PCR was performed using SYBR green mastermix (ThermoFisher Scientific) using EBNA-1 (EBNA1_F: TCATCATCATCCGGGTCTCC; EBNA1_R: CCT ACAGGGTGGAAAAATGGC); and β-actin (β-actin_F: GCCATGGTTGTGCCATTACA; β-actin_R: GGCCAGGTTCTCTTTTTATTTCTG) primers and relative EBV DNA copies were determined by ΔCt method.

### Chromatin Immunoprecipitation (ChIP)

ChIP was performed to determine EBNA1 binding to EBNA1/CTCF binding site using EBNA1 antibody (generated in-house in rabbits) as described previously [41]. ChIP DNA was assayed by qPCR using primers specific for indicated regions and quantified as % input. Primers for ChIP-qPCR are listed in S1 Table.

### RNA-seq

For all the samples, RNA was extracted and treated with DNase I using the DNase treatment kit (Ambion). RNA quality was determined using the Bioanalyzer (Agilent). Only samples with RIN numbers >7.5 were used for further studies. The total RNAseq libraries were made with KAPA Hyper-Prep with rRNA removal by using the Qiagen FastSelect kit, then subjected to 2 × 75-bp high-output sequencing on the Illumina NextSeq 500 platform.

The read quality was assessed using FASTX (http://hannonlab.cshl.edu/fastx_toolkit/) and FastQC (http://www.bioinformatics.babraham.ac.uk/projects/fastqc) and adapters were trimmed with Cutadapt (https://journal.embnet.org/index.php/embnetjournal/article/view/200). The resulting filtered reads were aligned using Bowtie2 [42] via RSEM V1.3.3 software [43]. Alignment was performed on a reference index created from the Ensemble transcriptome version GRCh37 reference genome for cellular genes and the NCBI Reference Sequence NC_007605.1 for EBV. This enabled estimation of read counts and RPKM values. Subsequently, a comprehensive quality report was generated using MultiQC [44]. Following this, in the read count QC stage, only the genes with ten or more reads in at least one sample were included. Samples were then assessed for outliers using Z-score, Principal Component Analysis (PCA), and correlation plots. Further, raw read counts of all genes were used as input to identify differentially expressed genes between groups using the R package DESeq2 [45]. Gene expression changes were considered significant if they passed the FDR < 0.05 threshold. With the significant genes, pathway analysis was performed with QIAGEN’s Ingenuity Pathway Analysis software (IPA, QIAGEN Redwood City, www.qiagen.com/ingenuity) using “Canonical pathways”, “Diseases & Functions”, and “Upstream Regulators” options to identify biological processes and signaling pathways significantly affected by the experimental conditions. Additionally, to obtain a holistic view of pathways associated with the entire gene set, Gene Set Enrichment Analysis (GSEA) [46] was also performed based on Gene Ontology (GO) terms, Molecular Signatures (MSigDB), Hallmark, Reactome, BioCarta, and KEGG pathway databases. Finally, for heatmap visualization of gene expression, we used DESeq2 normalized count values, and other plots were generated using the R package tidyverse (https://joss.theoj.org/papers/10.21105/joss.01686).

### Confocal imaging

Mutu I cells were treated with sodium butyrate and TPA for 48 h to induce lytic reactivation. Cells were washed with PBS and fixed with 4% (v/v) paraformaldehyde (ThermoFisher, J19943.K2) for 20 min at room temperature. Cells were then permeabilized in 0.2% Triton X-100 in PBS for 5 min and blocked with PBS containing 10% goat serum for 1 h at room temperature. Primary antibodies for CMPK2 (ThermoFisher, PA5–34461,1:50),TOM20 (Santa Cruz Biotechnology, sc-166755; 1:75), RSAD2 (Cell Signaling Technology, 13996, 1:100) and LMP1 (Millipore Sigma, MABF2248, 1:100) diluted in 1% goat serum were applied to the cells for 1.5 h at room temperature. After 3 washes with PBS containing 0.25% Tween 20, cells were incubated with Alexa 488-, Alexa 568-conjugated secondary antibodies (Invitrogen; 1:800) in 1% goat serum for 1 h at room temperature. Confocal imaging was performed on Leica TCS SP8 X WLL Scanning Confocal Microscope (Leica Microsystems). Images were acquired with 63x,1.4 NA oil immersion objective and the Leica LAS-X software. Confocal z-stacks were acquired with optimal z-intervals according to the Nyquist criterion, covering the whole volume of cells from the basal to the apical region. Images were then compiled by ‘max projection’ before analysis in ImageJ.

### Image analysis

Image analyses were conducted using ImageJ Fiji (v.2.9.0, National Institutes of Health). Maximum intensity projections were generated from z-stack confocal images. For the measurement of CMPK2 fluorescence intensity, intensity threshold segmentation was applied to CMPK2 channel to define region of interest (ROIs), and RawIntDen was calculated. For the measurement of CMPK2 colocalization with TOM20, intensity threshold segmentation was applied to individual cells to identify areas corresponding to CMPK2 and TOM20. Colocalization was assessed using the “JACoP” plugin in ImageJ to calculate Pearson’s coefficient. At least 25 cells randomly selected and pooled from 3 independent experiments were analyzed.

### Luciferase assay for NF-κB activity

One day prior to transfection, 293T cells were plated in 96-well plate. Next day, cells were transfected with plasmids pCMV-Flag, pcDNA3.1, pLMP1 (WT LMP1 cloned in pCMV-Flag), pRSAD2 (WT RSAD2 cloned in pcDNA3.1) or pNF-κB-Luc (5xNF-κB Luciferase pGL3 reporter) using Fugene transfection reagent (Promega). Twenty-four hours after transfection, expression of luciferase was determined using Luciferase Assay (Promega) as per manufacturer’s instructions. Relative luminescence units (RLU) were measured using CLARIOstar Plus microplate reader (BMG Labtech). For each transfection condition, two biological replicates were tested.

### Global metabolite profiling and targeted analysis of ddhCTP, ddhCDP and ddhCMP

RSAD2 and CMPK2 were knocked down in Mutu I cells followed by treatment with sodium butyrate and TPA for 24 h to induce lytic reactivation as described before. Cells were treated with 10 ng/ml IFN-α2A for 24 h to induce RSAD2 and CMPK2 expression and were used as positive control. After all treatments, cells were collected and polar metabolites were extracted with ice-cold 80:20 (v/v) methanol/water. For both global and targeted analyses, samples were analyzed by liquid chromatography- mass spectrometry (LC-MS) on a Thermo Scientific Q Exactive Plus mass spectrometer with HESI II probe in-line with a Thermo Scientific Vanquish UHPLC System. Samples were analyzed in a pseudorandomized order. LC separation was performed under HILIC conditions using a ZIC-pHILIC column (150 × 2.1 mm, 5 μm) maintained at 45 °C (EMD Millipore). Mobile phase A was 20 mM ammonium carbonate, 5 µM medronic acid, 0.1% ammonium hydroxide, pH 9.2, and mobile phase B was acetonitrile. Analytical separation was performed at 0.2 ml/min flow rate using the following gradient: 0 min, 85% B; 2 min, 85% B; 17 min, 20% B; 17.1 min, 85% B; and 26 min, 85% B. Specific MS parameters include: sheath gas, 30; auxiliary gas, 5; sweep gas, 0; auxiliary gas heater temperature, 200°C; spray voltage, 3.6 kV for positive/negative polarities; capillary temperature, 325°C; S-lens RF, 65.

For global metabolite profiling, samples were analyzed by either full MS scans with polarity switching (all samples) or full MS/data-dependent MS/MS scans with separate acquisitions for positive and negative polarities (sample pool). Full MS scans were acquired using a scan range of 65–975 m/z; 70,000 resolution; automated gain control (AGC) target of 1E6; and maximum injection time (IT) of 100 ms. Data-dependent MS/MS was performed on the 10 most abundant ions; 17,500 resolution; AGC target of 5E4; maximum IT of 50 ms; isolation width of 1.0 m/z; and stepped normalized collision energy of 20, 40, 60. Raw data were processed using Compound Discoverer 3.3 SP3 (Thermo Scientific) with separate analyses for positive and negative polarities. Metabolites were identified by matching accurate mass and retention time to analytical standards or querying MS/MS spectra against the mzCloud spectral database (full match, score > 50; mzCloud.org). Only [M + H]+ and [M-H]- adducts were considered for annotations. Metabolite levels were determined by integrated peak areas using Full MS data. Metabolite levels were corrected for instrument drift using peak areas from technical injections of the sample pool run periodically throughout the analysis and were subsequently normalized to total signal from annotated metabolites in each sample.

For targeted analysis of ddhCTP, ddhCDP, and ddhCMP, samples were analyzed by selective ion monitoring (SIM) mode in negative ion mode with an inclusion list (5 ppm mass tolerance) with the theoretical masses for [M-H]- adducts for ddhCTP (463.9667 m/z), ddhCDP (384.0003 m/z), ddhCMP (304.0340 m/z); 70,000 resolution; AGC target of 1E6; maximum IT of 50 ms; and isolation width of 1.0 m/z. Raw data were analyzed using TraceFinder 4.1 (Thermo Scientific). The peak detected for ddhCTP at 11.5 min was confirmed with a reference compound, while the peaks detected for ddhCDP at 10.8 min and ddhCMP at 9.6 min were unconfirmed, since no reference standard was available, but corresponded to the expected elution order of these compounds. Relative quantification was based on integrated peak areas, which were normalized to cell counts for each sample.

### Pathway enrichment analysis for global metabolite profiling

Pathway enrichment analysis for the global metabolite profiling data was performed using the Enrichment Analysis module in MetaboAnalyst 6.0 [47]. Significantly changed metabolites for a given comparison were defined as absolute fold-change greater than 1.5 and Benjamini-Hochberg adjusted p-value (q-value) of less than 0.05. The analysis was performed using the SMPDB metabolite sets, and the reference metabolome corresponded to the set of metabolites annotated in the global metabolite profiling analysis.

## Supporting information

S1 Fig RSAD2 is induced by NaB + TPA in EBV^+^ and EBV^-^ B-cells. A) EBV^+^ Akata cells were untreated or treated with NaB + TPA for 24 hrs and assayed by RT-qPCR for EBNA1, EBNA2, ZTA, RTA, EA-D, IFN-β, or RSAD2 relative to GUSB. B) LCL352 was untreated or treated with NaB + TPA for 24 hr and assayed by RT-qPCR for IFN-β (left) or RSAD2 (right) relative to GUSB. C) Western blot of EBV- BJAB or EBV+ Mutu I cells treated with NaB + TPA for 24 or 48 hrs and probed with antibody for RSAD2, EA-D, or β-Actin. Statistical analysis was performed using student’s two-tailed t-test. n = 3. * p < .05, ** p < .01, ***p < .001.
(TIF)

S2 Fig RNA-seq analysis of Mutu I responses to lytic induction. A) Volcano plot showing genes with most significant change in expression during NaB + TPA induction in Mutu I. B) Pathways analysis of genes upregulated by NaB + TPA in Mutu I cells. C-D) GSEA analysis of 3 most upregulated (C) and downregulated (D) hallmarks.
(TIF)

S3 Fig Differentially regulated genes in uninduced Mutu I cells after RSAD2 or CMPK2 knockdown. A) Top upregulated genes after RSAD2 knockdown uninduced Mutu I. B) Top downregulated genes after RSAD2 knockdown uninduced Mutu I. C) Top upregulated genes after CMPK2 knockdown in uninduced Mutu I. D) Top upregulated genes after CMPK2 knockdown in uninduced Mutu I.
(TIF)

S4 Fig Differentially regulated genes in induced Mutu I cells after RSAD2 or CMPK2 knockdown. A) Top upregulated genes after RSAD2 knockdown for induced Mutu I. B) Top downregulated genes in RSAD2 knockdown for induced Mutu I. C) Top upregulated genes after CMPK2 knockdown in induced Mutu I. D) Top downregulated genes after CMPK2 knockdown in induced Mutu I.
(TIF)

S5 Fig RT-qPCR validation studies for RNA-seq. RT-qPCR validation for CXCL10, CCL22, IFI-6, and RUNX1T1 in shRSAD2 (A) or shCMPK2 (B) transduced Mutu I cells during lytic induction. Statistical analysis was performed using student’s two-tailed t-test. n = 3. * p < .05, ** p < .01, ***p < .001.
(TIF)

S6 Fig Detection of ddhCMP and ddhCDP in Mutu I by mass spectrometry. Using cell extracts as described in Fig 7B, abundance of ddhCMP (A) and ddhCDP (B) were measured in Mutu I cells treated with shCtrl, shCMPK2, or shRSAD2, in uninduced or induced Mutu I, or compared to IFN-α2A treatment.
(TIF)

S7 Fig Metabolomic analyses for knockdown in uninduced Mutu I and after IFN-α2A treatment. A) Top 10 up/downregulated metabolites after RSAD2 knockdown in uninduced Mutu I. B) Pathway enrichment analysis for RSAD2 knockdown (uninduced). C) Top 10 up/downregulated metabolites after CMPK2 knockdown in uninduced Mutu I. D) Pathway enrichment analysis for CMPK2 knockdown (uninduced). E) Top 10 up/downregulated metabolites after IFN-α2A treatment. F) Pathway enrichment analysis for IFN-α2A treatment.
(TIF)

S1 Table Oligonucleotides used for RT-qPCR and ChIP-qPCR. (PDF)

S1 Data Metabolomics data for Mutu I cells treated with IFN-α2A or transduced with shCtrl, shRSAD2 or shCMPK2 under uninduced latency or lytic EBV reactivation. (XLSX)

S1 Raw Gels Uncropped Western blot images for Figs 1E,1F, 2B, 3F,3I, 4C,4D, 6C and 7D,7F. Cropped images are indicated with red boxes.
(PDF)
