# Supplementary material for: RSAD2/VIPERIN and CMPK2 coordinate an immunometabolic response to Epstein-Barr Virus
Source: PLoS Pathog. 2026 Aug 3;22(8):e1013973. doi: 10.1371/journal.ppat.1013973 (PMC13456488; doi:10.1371/journal.ppat.1013973)
Supplement: Supplementary file 1 [file ppat.1013973.s008.pdf]

Table S1

Assay	Gene	Primer	Sequence
RT-qPCR	EBNA1	Forward	GGTCGTGGACGTGGAGAAAA
		Reverse	GGTGGAGACCCGGATGAT G
	EBNA2	Forward	CCAGCGCCAATCTGTCTACA
		Reverse	TGATGGCGGCAGATAAAGC
	LMP-1	Forward	TCCAGAATTGACGGAAGAGGTT
		Reverse	GCCACCGTCTGTCATCGAA
	ZTA	Forward	TCTGAACTAGAAATAAAGCGATACAAGAA
		Reverse	TTGGGCACATCTGCTTCAAC
	EA-D	Forward	TTGGGCAGGTGCTGTTGAT
		Reverse	TGCCCACTTCTGCAACGA
	EBER-1	Forward	TTTGCTAGGGAGGAGACGTGTGT
		Reverse	AAGCAGAGTCTGGGAAGACAACCA
	RSAD2	Forward	TGCTTTTGCTTAAGGAAGCTG
		Reverse	CAGGTATTCTCCCCGGTCTT
	CMPK2	Forward	CCAGGTTGTTGCCATCGAAGGA
		Reverse	GGTGGTGACTTTAAGAGGACAGC
	GUSB	Forward	CGCCCTGCCTATCTGTATTC
		Reverse	TCCCCACAGGGAGTGTGTAG
ChIP-qPCR	EBV-FR	Forward	CAGCGACCTCGTGAATATGA
		Reverse	AAACCACTTGCCCACAAAAC
	EBV-OriLyt	Forward	TCGCCTTCTTTTATCCTCTTTTTG
		Reverse	CCCAACGGGCTAAAATGACA
	EBNA1 binding site	Forward	AGTGTGTCAGGATAGCACCC
		Reverse	ACTGCGCAAACACTGTTACAC
