# Supplementary material for: RSAD2/VIPERIN and CMPK2 coordinate an immunometabolic response to Epstein-Barr Virus
Source: PLoS Pathog. 2026 Aug 3;22(8):e1013973. doi: 10.1371/journal.ppat.1013973 (PMC13456488; doi:10.1371/journal.ppat.1013973)

Original Western Blot Images

Figure 1B

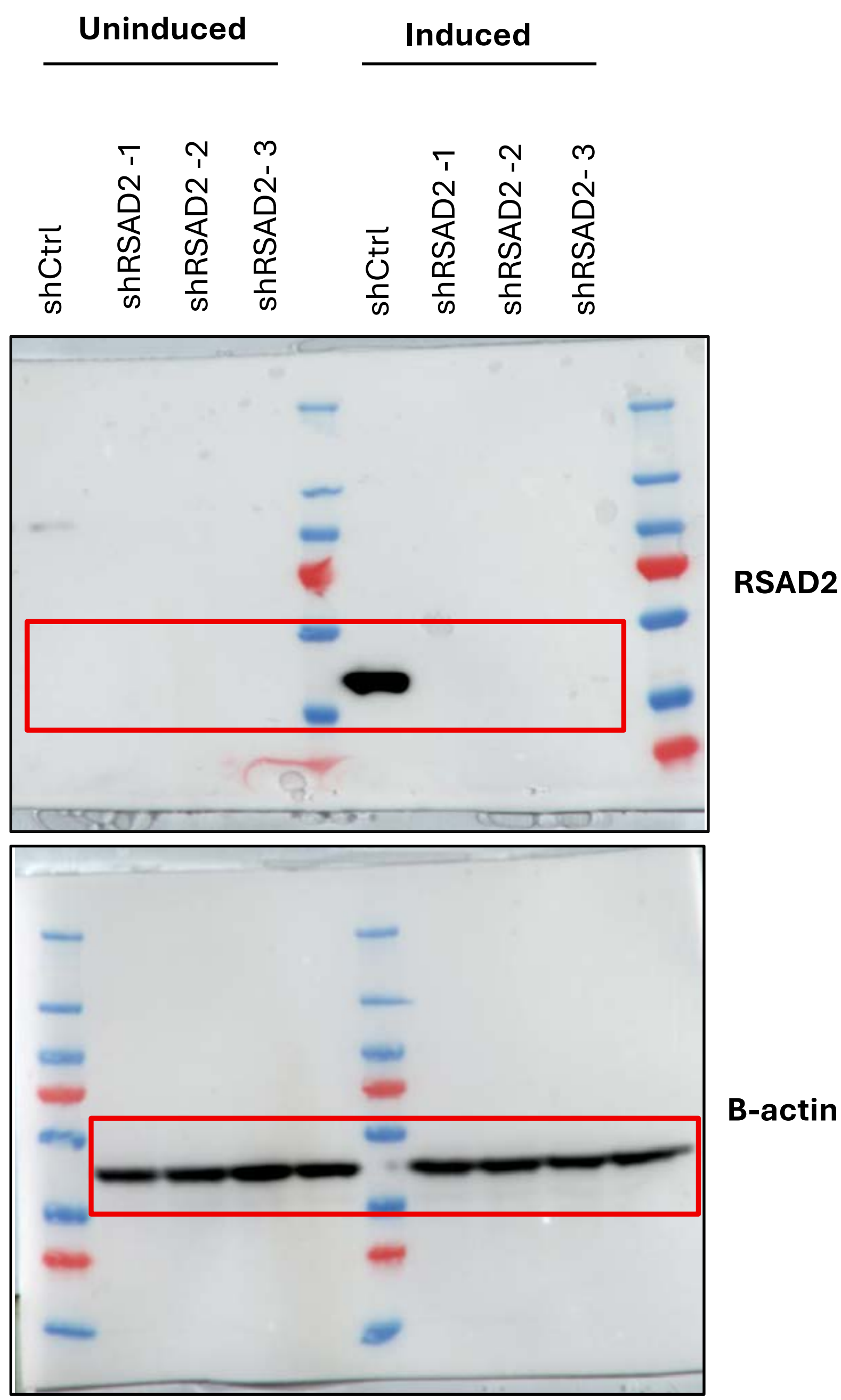

Figure 1F

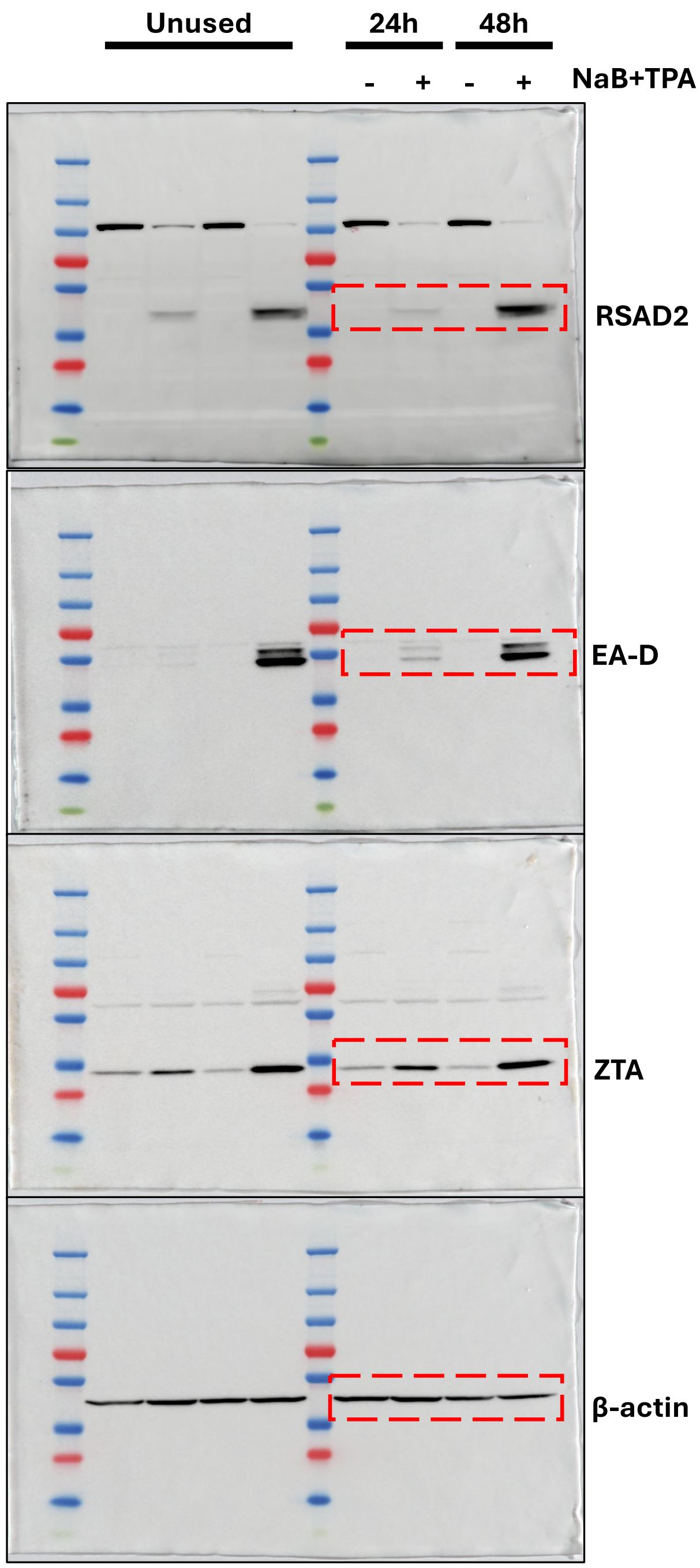

Figure 1G

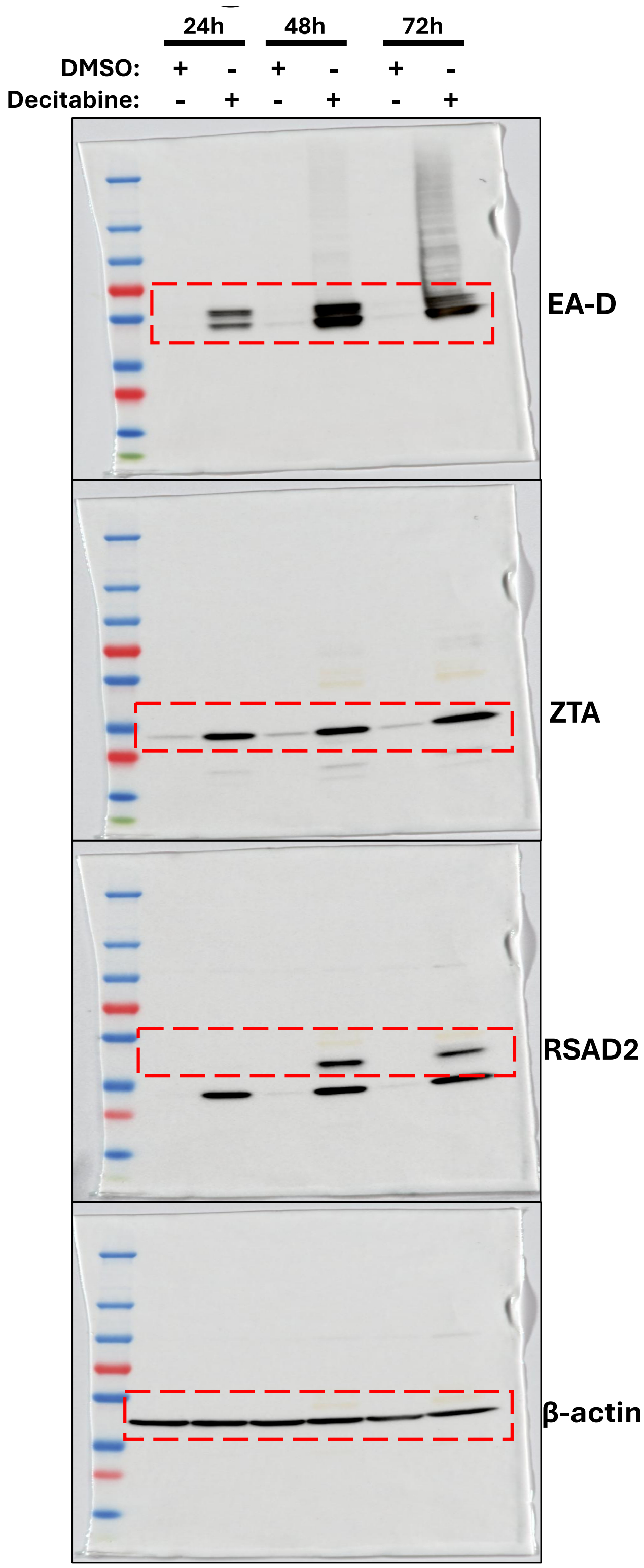

Figure 3F

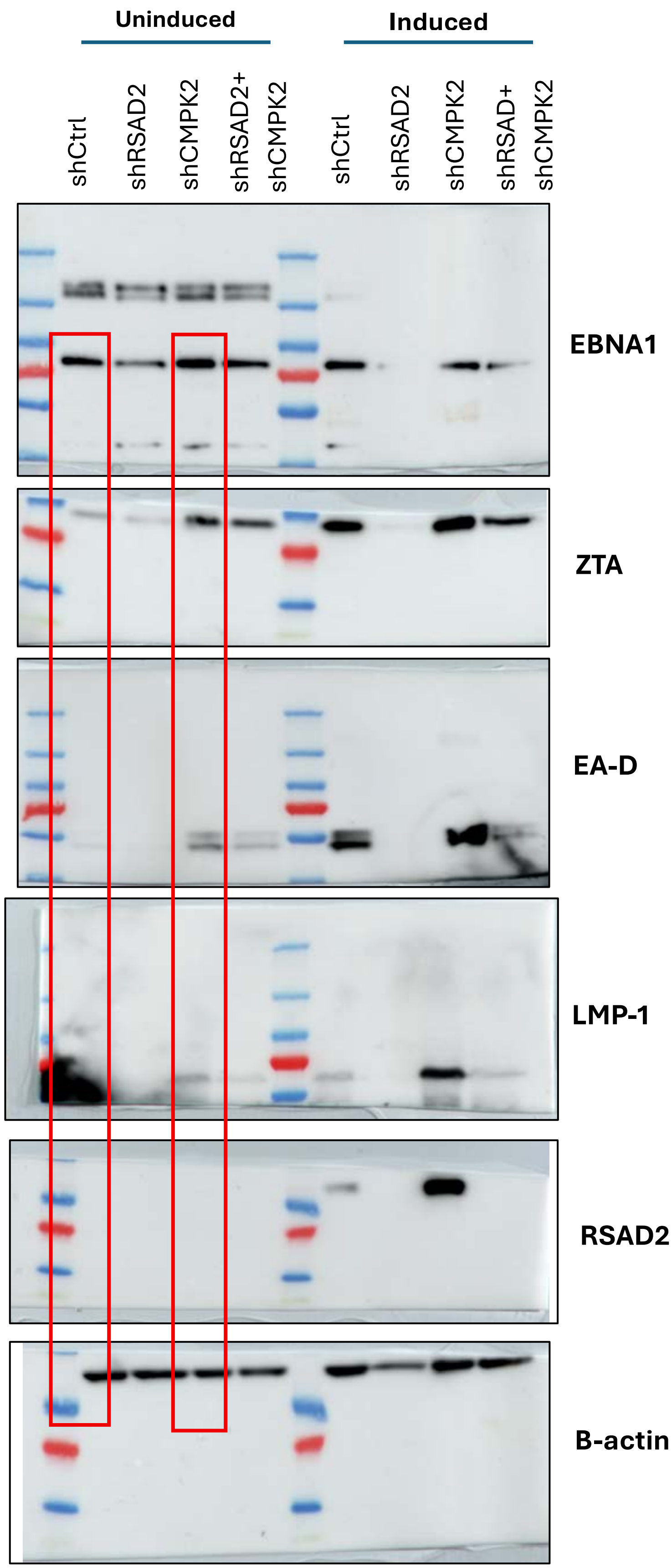
Uninduced
Induced
shCtrl
shRSAD2
shCMPK2
shRSAD2+
shCMPK2
shCtrl
shRSAD2
shCMPK2
shRSAD+
shCMPK2
EBNA1
ZTA
EA-D
LMP-1
RSAD2
B-actin

Figure 3I

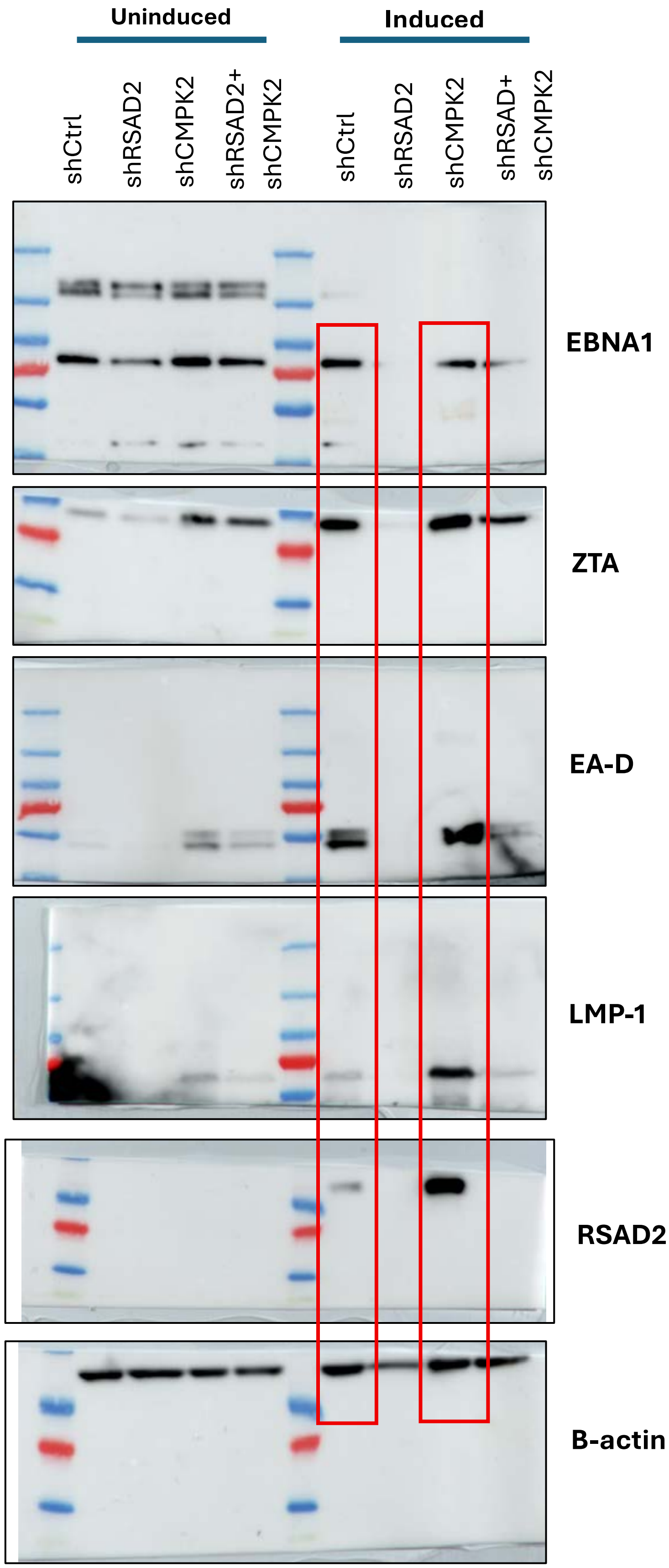
Uninduced
Induced
shCtrl
shRSAD2
shCMPK2
shRSAD2+ shCMPK2
shCtrl
shRSAD2
shCMPK2
shRSAD+ shCMPK2
EBNA1
ZTA
EA-D
LMP-1
RSAD2
B-actin

Figure 4C

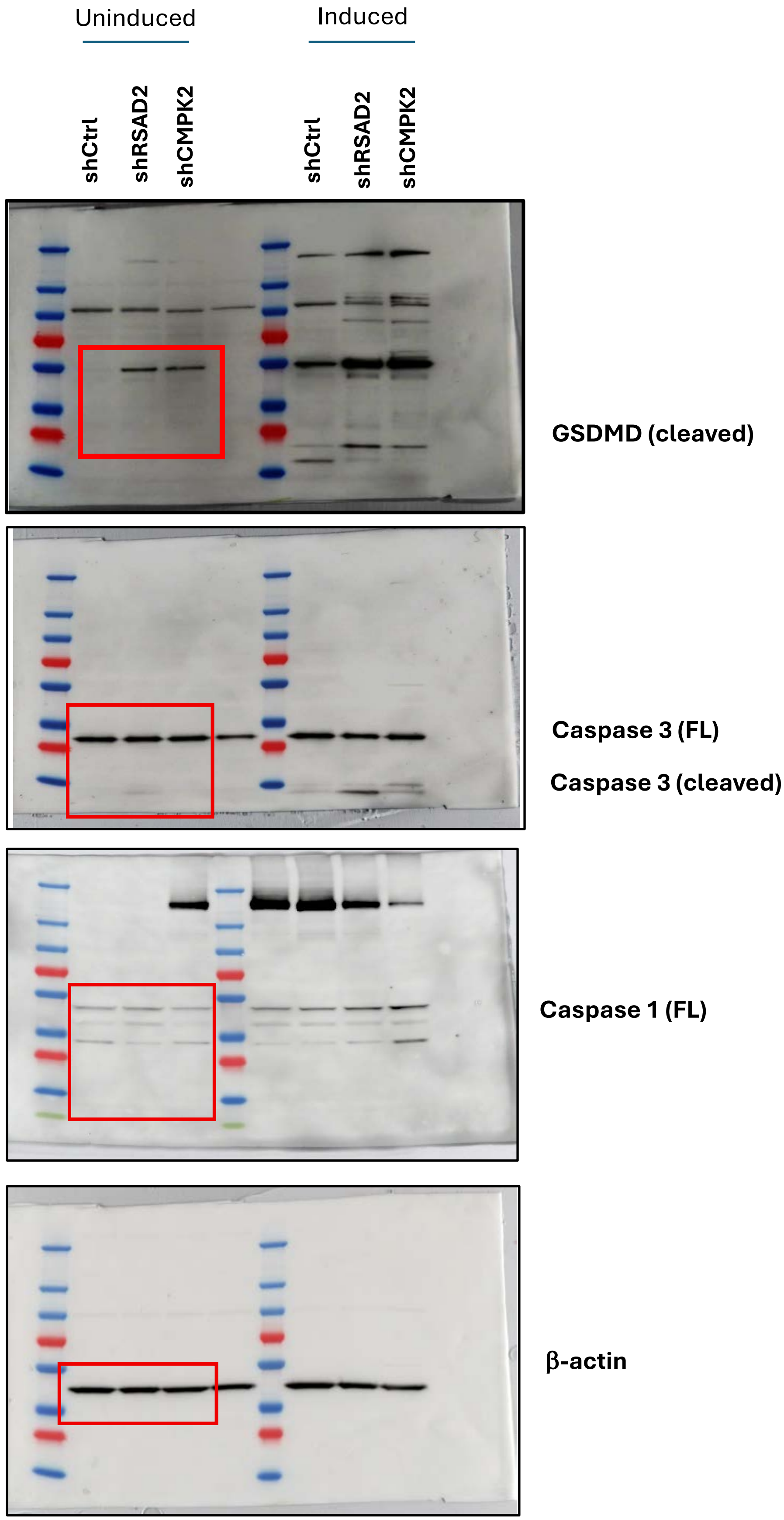
Uninduced
Induced
shCtrl
shRSAD2
shCMPK2
shCtrl
shRSAD2
shCMPK2
GSDMD (cleaved)
Caspase 3 (FL)
Caspase 3 (cleaved)
Caspase 1 (FL)
β-actin

Figure 4 D

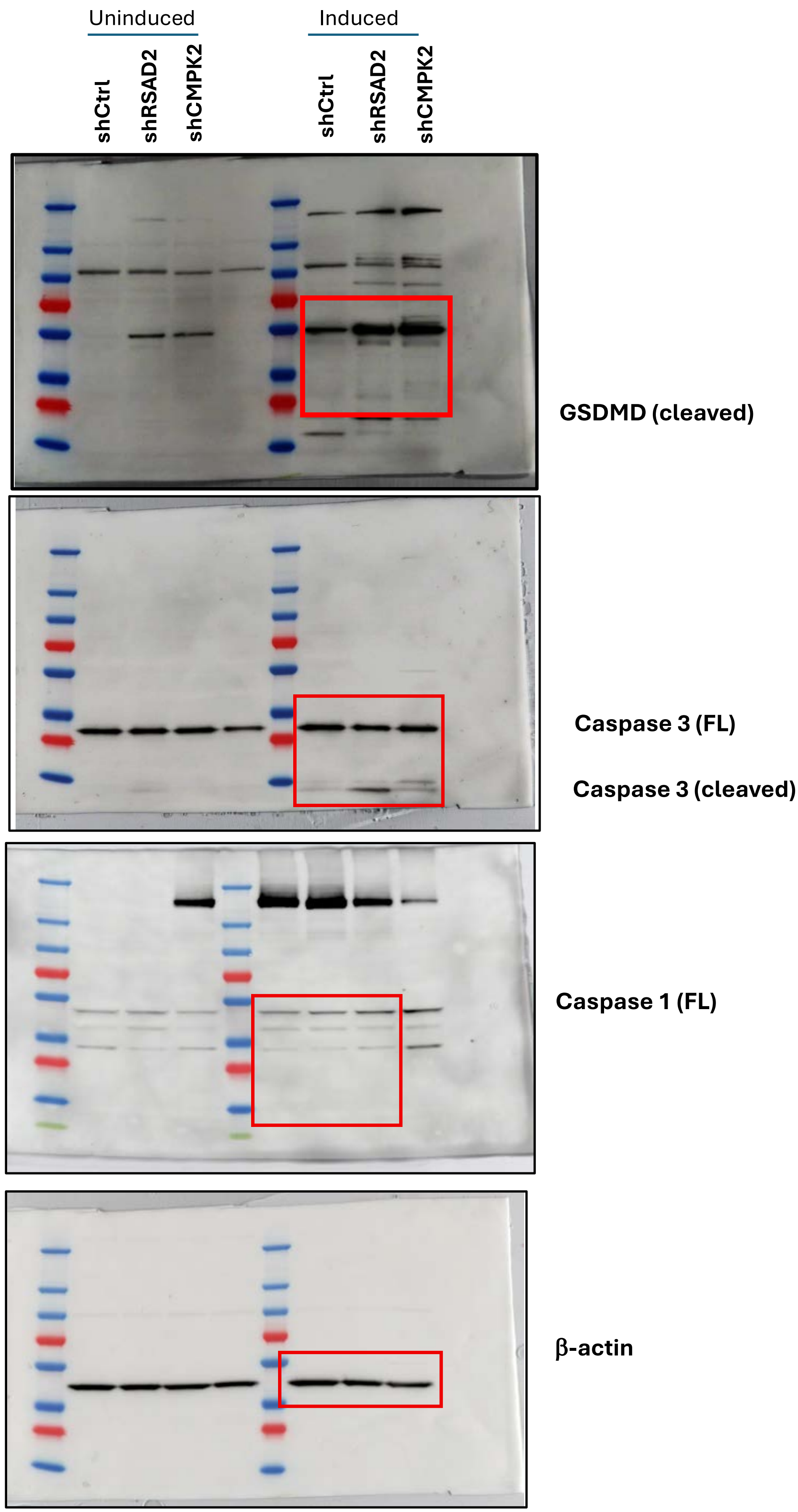

Uninduced
Induced
shCtrl
shRSAD2
shCMPK2
shCtrl
shRSAD2
shCMPK2
GSDMD (cleaved)
Caspase 3 (FL)
Caspase 3 (cleaved)
Caspase 1 (FL)
β-actin

Figure 6C

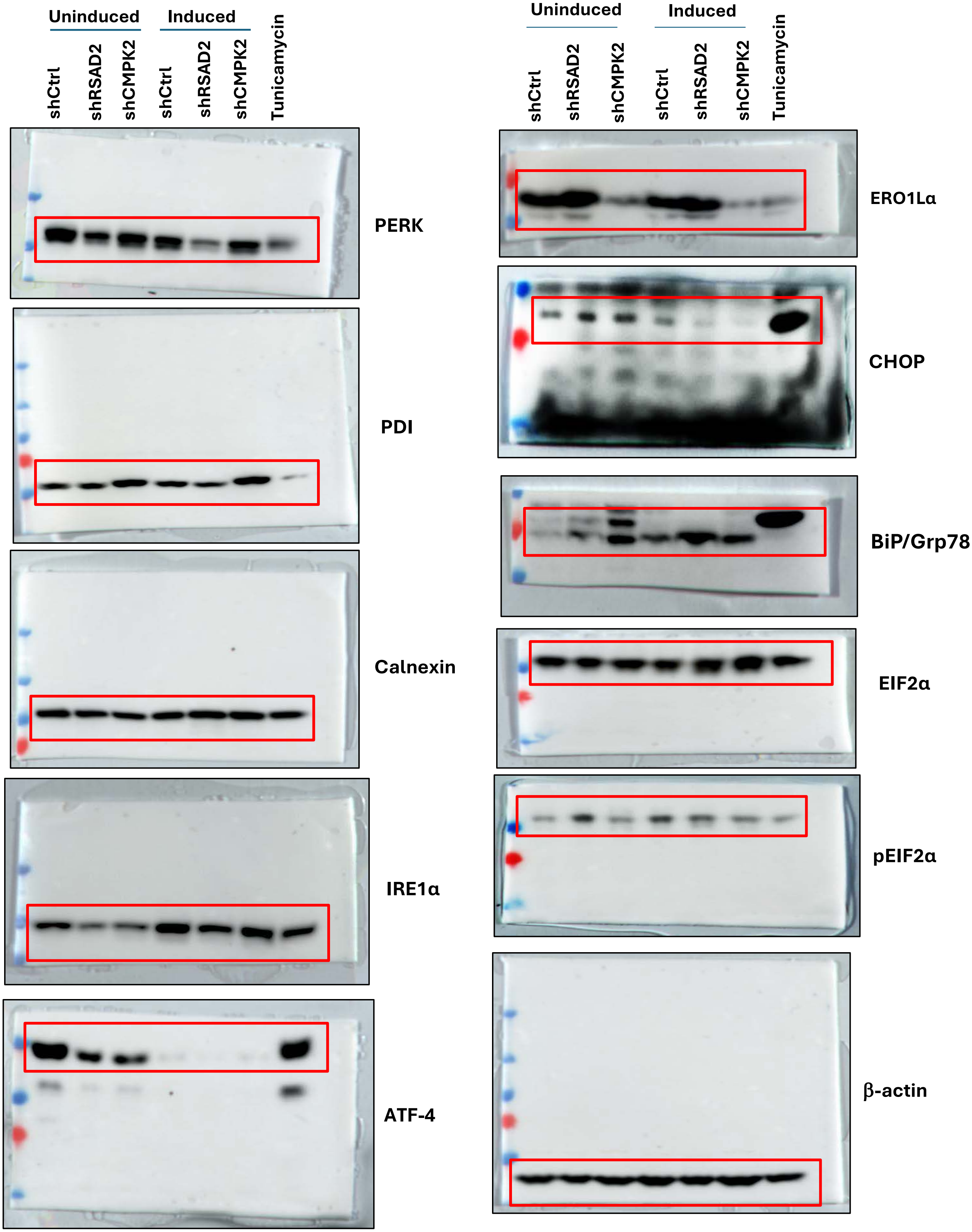
Uninduced
Induced
shCtrl
shRSAD2
shCMPK2
shCtrl
shRSAD2
shCMPK2
Tunicamycin
PERK
PDI
Calnexin
IRE1α
ATF-4
Uninduced
Induced
shCtrl
shRSAD2
shCMPK2
shCtrl
shRSAD2
shCMPK2
Tunicamycin
ERO1Lα
CHOP
BiP/Grp78
EIF2α
pEIF2α
β-actin

Figure 7D

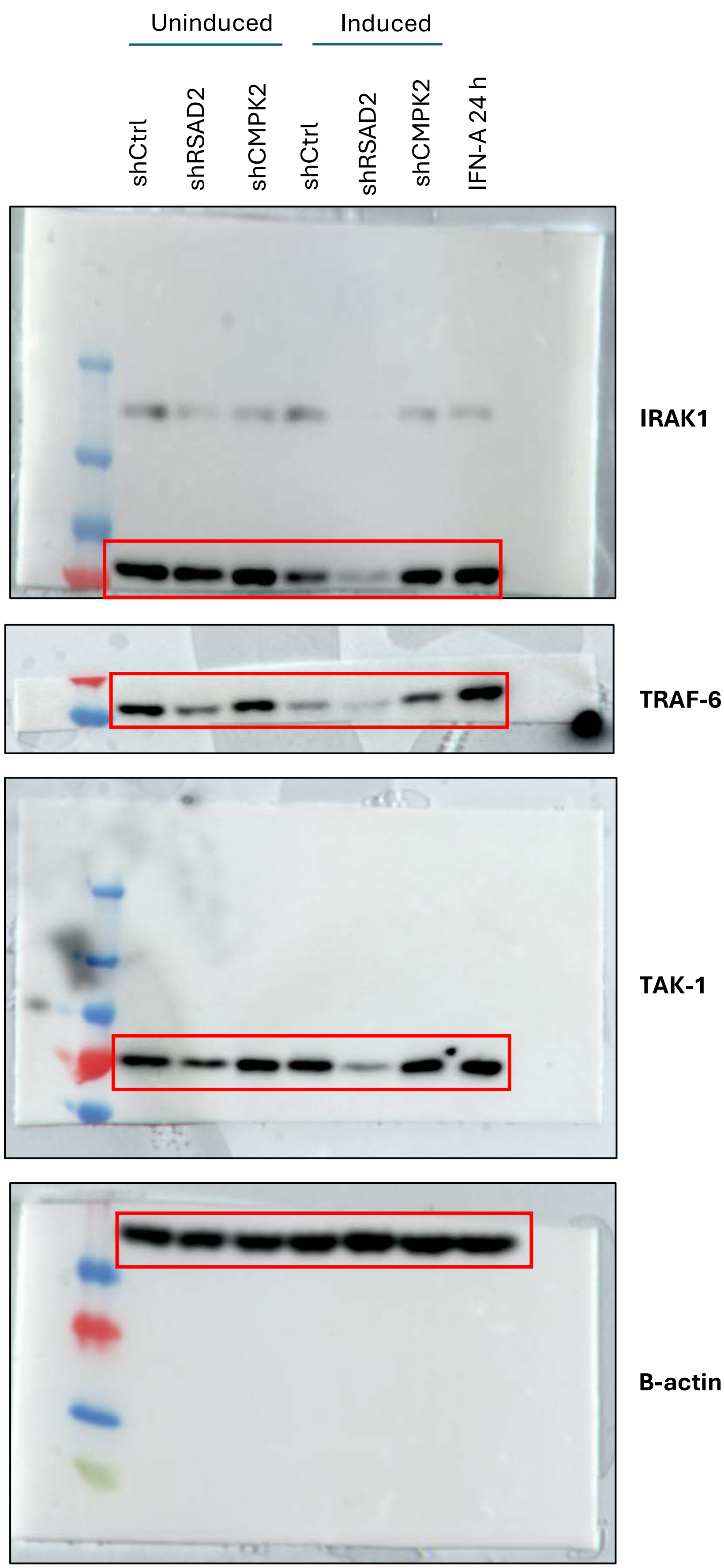

Uninduced
Induced
shCtrl
shRSAD2
shCMPK2
shCtrl
shRSAD2
shCMPK2
IFN-A 24 h
IRAK1
TRAF-6
TAK-1
B-actin

Figure 7F

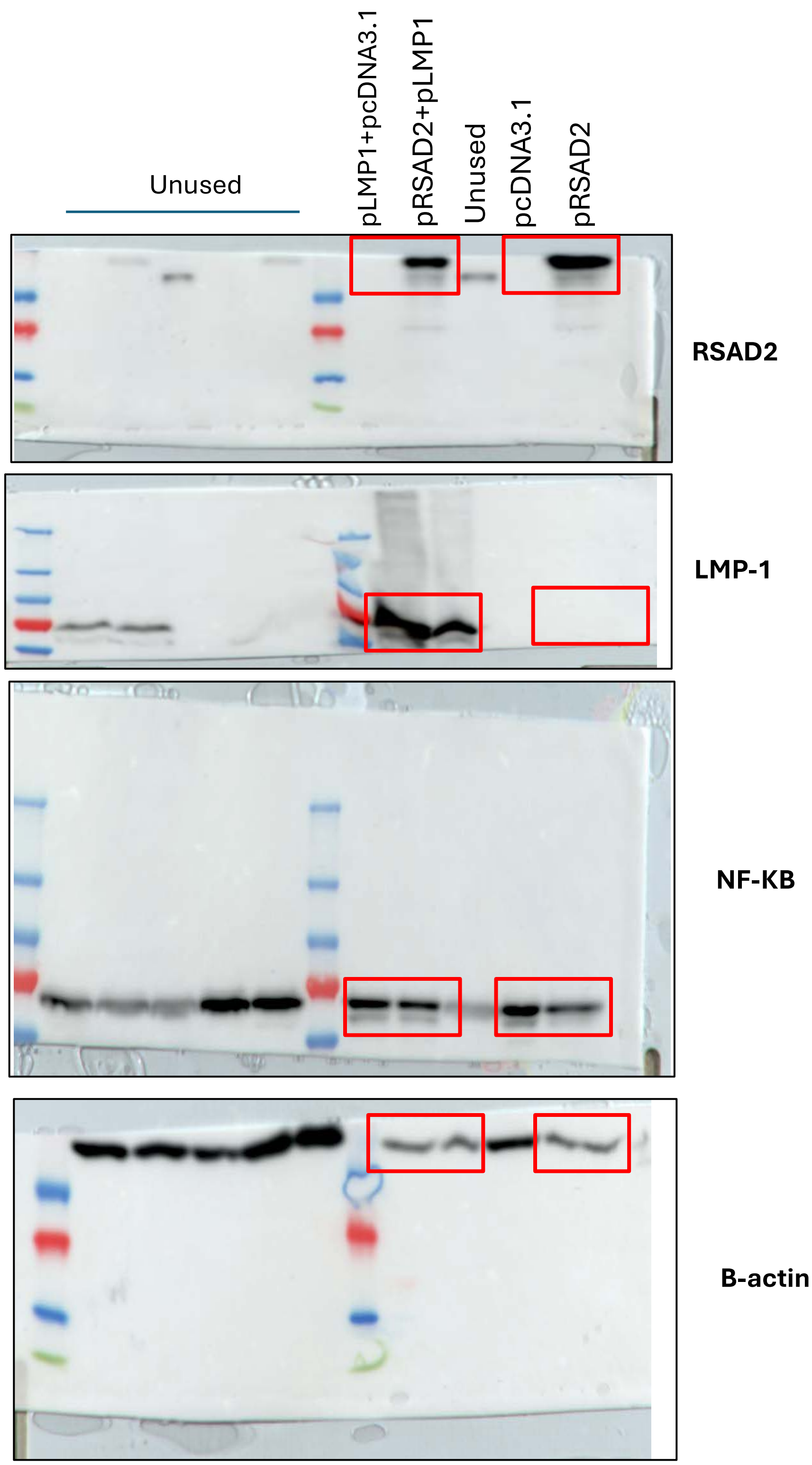

Unused
pLMP1+pcDNA3.1
pRSAD2+pLMP1
Unused
pcDNA3.1
pRSAD2
RSAD2
LMP-1
NF-KB
B-actin

Figure S1C

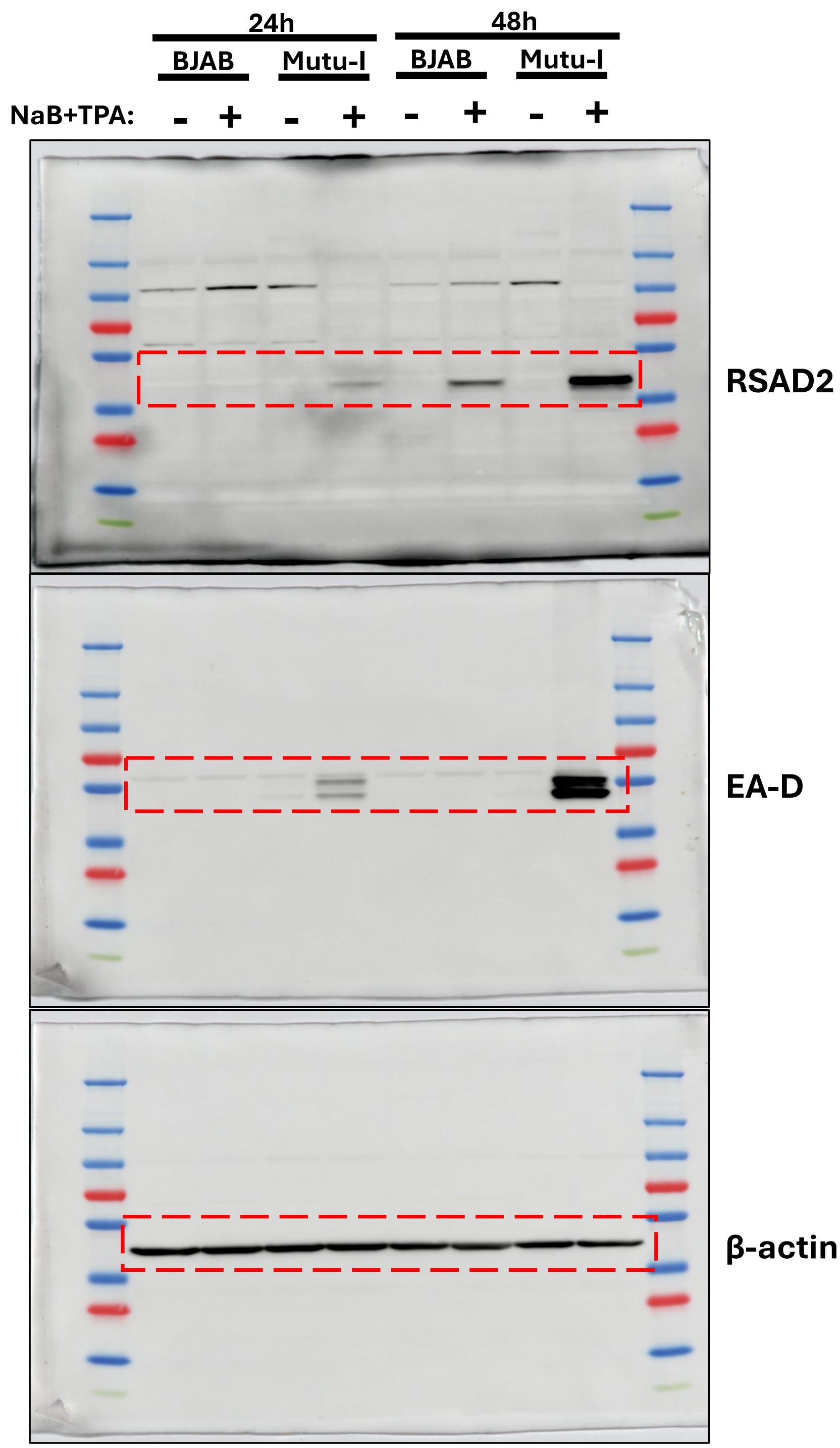

Supplement: Supplementary file 2 [file ppat.1013973.s010.pdf]
